# A Survey on Detection, Tracking and Identification in Radio Frequency-Based Device-Free Localization

**DOI:** 10.3390/s19235329

**Published:** 2019-12-03

**Authors:** Stijn Denis, Rafael Berkvens, Maarten Weyn

**Affiliations:** IDLab–Faculty of Applied Engineering, University of Antwerp–imec, Sint-Pietersvliet 7, 2000 Antwerp, Belgium; rafael.berkvens@uantwerpen.be (R.B.); maarten.weyn@uantwerpen.be (M.W.)

**Keywords:** localization, device-free, radio frequency, RF, wireless sensor networks, WSN, radio tomographic imaging, RTI, passive identification, passive tracking, passive detection, sensorless sensing

## Abstract

The requirement of active localization techniques to attach a hardware device to the targets that need to be located can be difficult or even impossible for certain applications. For this reason, there has been an increasing interest in tagless or device-free localization (DFL) approaches. In particular, the research domain of RF-based device-free localization has been steadily evolving since its inception slightly over a decade ago. Many novel techniques have been developed regarding the three core aspects of DFL: detection, tracking, and identification. The increasing use of channel state information (CSI) has contributed considerably to these developments. In particular, the progress it enabled regarding the exceptionally difficult ‘identification problem’ has been highly impressive. In this survey, we provide a comprehensive overview of this evolutionary process, describe essential DFL concepts and highlight several key techniques whose creation marked important milestones within this field of research. We do so in a structured manner in which each technique is categorized according to the DFL core aspect it emphasizes most. Additionally, we discuss current blocking issues within the state-of-the-art and suggest multiple high-level research directions which will aid in the search towards eventual solutions.

## 1. Introduction

Over the last few decades, a large amount of research has been performed regarding the automatic localization of both human and non-human entities. GPS [1] has become a ubiquitous technology in the context of outdoor navigation, while many different solutions exist for indoor applications. Active infrared, ultrasonic sound waves, radio frequency (RF) waves [2] and visible light [3] are but a few examples of technologies that have been extensively studied for this purpose. Furthermore, it is no longer necessary in the deployment of systems utilizing these technologies to make use of high-cost, specialized hardware [4]. In certain applications, common consumer-grade items like smartphones and laptops can be used in combination with an existing network infrastructure (e.g., a set of Wi-Fi access points) to achieve a high level of localization accuracy. With the advent of smart cities and the Internet of Things [5], this ease of deployment is likely to increase even further.

The majority of these systems require that the entity that needs to be located is wearing or is attached to a passive or active hardware device: a tag. In other words, it is the tag itself whose location is actually determined due to its transmission of or response to a certain type of signal. There are a number of applications where this requirement can be highly problematic. The use of localization technologies in elderly care or assisted living situations is one such example. Always carrying a tag (regardless of the form it takes) can be perceived as stigmatizing and the residents of an elderly care institution could inadvertently forget to take it with them. Other examples include applications where people in danger need to be located quickly by emergency services or security systems that are capable of both detecting and tracking intruders. In these cases, it is impossible to tag the entities beforehand.

For this reason, there has been an increasing interest in device-free or tagless localization. These techniques do not require entities to wear any kind of device whatsoever and instead make use of the influence the physical presence of an entity has on its environment to determine its location.

Youssef et al. [6] were amongst the first to formally outline the field of device-free (passive) localization (DFL). They described DFL as consisting of three core aspects: detection, tracking, and identification.

Detection refers to the ability of a tagless localization system to detect if there are any changes in an environment caused by non-static entities and if so, estimate how many entities are causing these changes.

Tracking is to be interpreted as the continuous estimation of positions and velocities of the detected entities. This aspect tends to be focused on the most in DFL systems.

Finally, identification refers to the system’s ability to determine the identity of the non-static entities. Identity in this context is a loosely interpretable term and can refer to the size, shape, material or type of an entity or to an actual human identity. Solving the ‘identification problem’ with a non-camera based solution is still considered to be a major challenge in this field of research and only in recent years have there been some successful attempts [7,8,9]. Interestingly, this difficulty (or depending on the technique, outright impossibility) of correctly identifying a target can be considered an advantage for applications where the aspect of privacy is paramount.

Many different types of tagless localization techniques already do exist, each with their own strengths and weaknesses based on the type of sensor they use. Optical multi-camera based techniques are a popular type of device-free localization and they are capable of accurately tracking people in a complex indoor environment [10]. Furthermore, correctly identifying the targets through the use of computer vision-techniques is much easier than for other types of DFL, even if low-resolution camera images are used [11]. They tend to require a large amount of computing power, however, and their reliability can be severely impacted by environmental factors such as lighting conditions and potential occlusions [12]. Additionally, as stated in the previous paragraph, the ability to identify entities is a double-edged sword and will always raise the specter of privacy-related issues. Another type of DFL is passive infrared localization [13], which makes use of the thermal radiation emitted by human bodies. While effective and easily implementable through the use of relatively few low-cost sensors, its performance suffers severely in non-line of sight situations. Other systems exist based on floor vibrations [14], differential air pressure [15] and capacitance between floor tiles and electrodes [16]. All of these systems are but a few examples of the different types of tagless localization that have been developed.

One very interesting approach towards DFL consists of analyzing the influence a target has on radio frequency (RF) communication within the environment. Through-the-wall imaging systems based on measuring the reflection of transmitted ultra-wideband signals are commercially available by companies such as Camero Tech [17], whose products are specifically focused on police- and military-related applications. Two important disadvantages of these radar-like systems are the high cost of the specialized hardware and their accuracy-related problems at large distances [18], however. Another reflection-based technique is MIMO-radar, which has attracted investigative interest for human localization in complex indoor environments [19,20,21].

A potential alternative to these techniques is to focus on measuring the transmission between a network of RF nodes, rather than a reflection of the signal. Many techniques incorporating this principle are able to use low-cost off-the-shelf hardware or—in a similar vein to certain aforementioned tagged localization techniques—can even implement their entire system on existing network infrastructure.

Whether existing infrastructure is used or not, the network of nodes whose RF measurements are analyzed is a wireless sensor network in which the radios themselves—usually only used for the communication of sensor values—are actually the sensors. For this reason, these types of networks are called RF sensor networks. Another term that is often used to denote technologies that make use of this principle is ‘sensorless sensing’. Interest in the concept itself of using radios as sensors has been increasing and a general overview can be found in [22]. This interest is not solely limited to applications that are strictly related to localization. Other research domains such as device-free activity recognition [23,24,25,26,27], health monitoring (e.g., breathing monitoring) [28,29], gesture recognition [30,31] and fall detection [32] have become popular topics of research as well. The use of these technologies in the context of smart homes and assisted living for the elderly has attracted particular attention [33].

**Figure 1:**
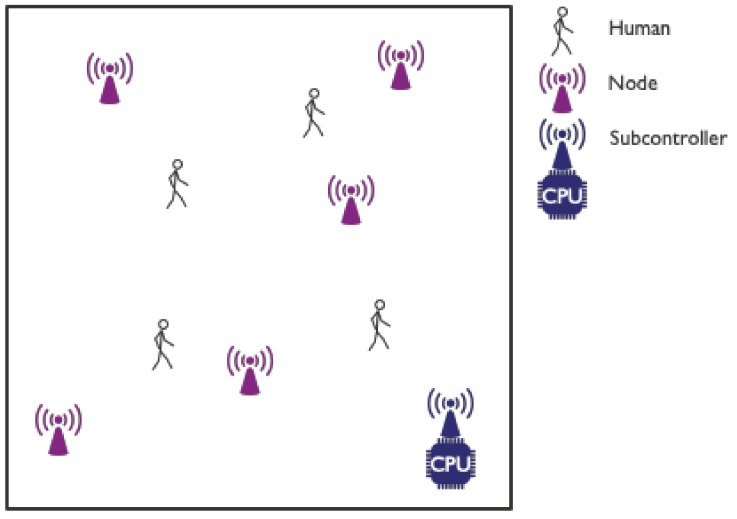
A basic device-free localization (DFL) system.

A schematic overview of the most common interpretation of the DFL concept is provided in Figure 1. Multiple human targets are present in an environment in which several RF nodes have been installed. Depending on the specific setup, these nodes can be transmitters, receivers or transceivers. One of the nodes acts as a (sub)controller node which regulates communication within the network and captures the desired characteristics from this RF communication. The captured data is sent to some type of processing unit (a laptop, a NUC, ...) where it is stored and/or used as input to a DFL algorithm. This algorithm will utilize the impact of the human presence on RF communication within the network for detection, tracking and/or identification of the targets.

In this paper, we perform an extensive survey of the literature regarding the use of RF sensor networks for device-free localization. Each DFL technique we describe is categorized based on which of the three aspects described by Youssef et al. [6] it focuses on most strongly: detection, tracking, or identification. While most DFL systems contain elements of two or even all three of these aspects, the (potential) applications of a technique often dictate where the focus lies. For each aspect, we discuss the evolution of the research field and make analytical comparisons between the different techniques that have been developed over the years.

It should be noted that many of the sensorless sensing fields of research mentioned previously are very closely related to DFL. As an example, human activity can potentially be linked directly to the location of the individual, as demonstrated in [24,25]). Activity recognition has become a particularly popular passive sensing topic in recent years, which is illustrated by the fact that it has been the subject of a two-part Feature Topic in IEEE Communications Magazine [26,27]. Nevertheless, our focus lies on localization and we consider these other research domains to be outside of the scope of this survey.

Despite the relative youth of the DFL field of research, several other survey and general overview papers have been created regarding (subsections of) this topic. An early overview from 2010 regarding the existing literature was given by Patwari et al. in [34]. Deak et al. discussed the use of DFL in a specific application domain: disaster management scenarios [35]. In 2013, Pirzada et al. compared several (at the time) popular DFL techniques that were both RF and non-RF-based [36]. Zhou et al. described the state-of-the-art regarding sensorless sensing based on Wi-Fi signals in [37]. Of particular interest here is the fact that they noticed an evolution from purely RSS-based techniques to techniques that also incorporate channel state information (CSI), an aspect which we will discuss in this paper as well. In [12], the state of the art of crowd estimation technologies, in general, is shown, which can be considered to be part of the detection aspect of DFL. This paper does not only discuss RF-based solutions but other techniques as well, both tagless (e.g., computer vision) and tagged (e.g., smartphone detection based on Bluetooth or Wi-Fi). Additionally, a fairly large overview of pre-2016 RF-based small-scale detection and tracking techniques with a pronounced focus on radio tomographic imaging (RTI) was provided by Shukri et al. in [38]. Finally, a comparatively recent and highly interesting broad overview of DFL-approaches was provided by Palipana et al. in [39]. While all these papers give interesting insights into specific aspects of RF-based DFL, none of them provides an in-depth overview and analysis of the evolution towards the current state-of-the-art which encompasses all three core aspects of the topic: detection, tracking, and identification. The main goal of this paper is to provide this overview in a comprehensive manner so that it may be used as a reference work by both novice and experienced researchers in this field.

The rest of this paper is structured as follows. In Section 2 we provide an overview of DFL techniques that are primarily focused on detection. A subdivision is made between relatively simple motion detection systems capable of detecting the presence of a limited number of intruders and crowd estimators capable of providing an estimate for the size and/or density of a large group of humans.

Section 3 contains information regarding tracking in tagless localization. An entire subsection is devoted to radio tomographic imaging (RTI) and its multitude of variants and improvements. RTI was first developed by Patwari et al. [18,40,41] and has become a mainstay in this field of research. Another subsection describes several other model-based device-free techniques that do not make use of an RTI imaging step. Finally, we also detail many techniques that make use of passive radio mapping (or passive fingerprinting). In contrast to its more well-known active equivalent, a passive radio map does not contain measurements regarding the communication between a tag and a set of fixed nodes when the target is at a certain location. Rather, it makes use of the communication between the fixed nodes themselves when a tagless entity is present.

Section 4 discusses the existing literature regarding RF-based tagless identification systems. Non-camera based solutions to the identification problem have been developed only very recently. This section is subdivided into a category for RSS-based fingerprinting approaches and a category for CSI-based approaches that allow for human differentiation based on gait analysis.

In Section 5 we describe potential future research directions for all three aspects of device-free localization. Finally, a conclusion to this work is presented in Section 6.

**Figure 2:**
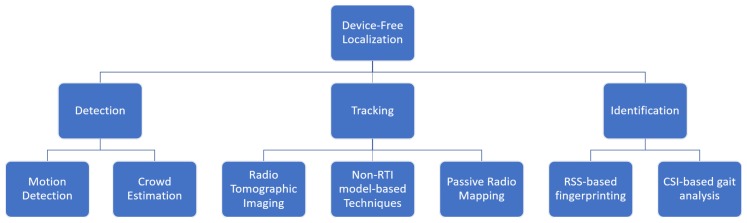
A schematic overview of the survey paper structure.

A schematic overview of the way this paper is structured can be found in Figure 2.

## 2. Detection

In this section, we will divide the detection techniques we describe into two main categories: techniques focused on motion detection and techniques focused on crowd estimation. Systems utilizing techniques from the first category solely attempt to determine if there are moving entities within the environment that are influencing RF communication within a WSN. Systems utilizing techniques from the second category also want to know how many different entities are causing these changes. This second category will contain an overview of both detection methods which aim to provide an exact count of the number of targets (crowd counting) and detection methods which abstractedly estimate how crowded an environment is (crowd density estimation).

**Figure 3:**
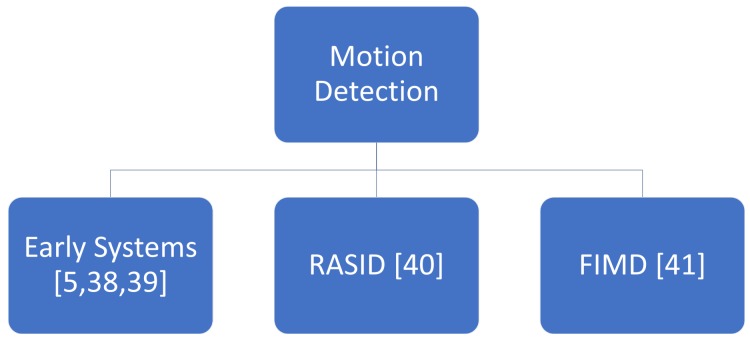
A schematic overview of the main motion detection approaches discussed in this survey.

A schematic overview of the main approaches we discuss in this survey for motion detection is provided in Figure 3.

### 2.1. Motion Detection

#### 2.1.1. Early Systems

Initially, we will describe several early motion detection systems that were developed during the first few years after the formal outlining of the field by Youssef et al. [6], beginning with the initial system they proposed in the very same paper to illustrate the feasibility of the concept. The system they built encompassed both detection and (limited) tracking capabilities for a single human intruder. In this section, we will discuss the detection aspect of this system and consider it to be a starting point for all detection-related DFL research that has been performed over the past decade. The fingerprint-based tracking method they used will be discussed in more detail in Section 3.

The detection system Youssef et al., constructed consisted of 2 IEEE 802.11b access points (AP) which periodically broadcasted packets and two wireless sniffers which acted as monitoring points (MP). In total, this meant that there were only four one-way communication links that could be used to detect potential changes in the environment. Each MP would send the RSS values with which it received the aforementioned packets to an application server (AS) on which algorithms for detection (and tracking) could be performed. Two different detection methods were investigated: moving average-based detection and moving variance-based detection. In the moving average-based detection method, events (i.e., changes in the environment, most likely caused by targets) were detected by calculating the differences between short-term and long-term moving averages of the RSS values of each link. The moving variance-based detection approach made use of the differences between the continuously updated moving variances and the average variances measured during calibration measurements when the environment was entirely static. When a difference calculation on a single link exceeded a certain predefined threshold, an event detection alert was raised. If a certain number of alerts were raised within a certain period of time referred to as the alert time buffer, an actual event detection for the entire system was declared. Through parameter optimization, both techniques managed to obtain a detection accuracy of 100% with 0 false positives.

It should be noted, however, that these accuracies were obtained in a very simple, controlled test environment. In [42], Moussa and Youssef continued their research and applied the same two detection methods to a similar setup in a 25 m^2^. square area within a university lab. Measurements were explicitly performed during the day when other people were present in the same building. Results showed a vast increase in false positives when compared to the more idealized environment, with precision ratios (correct event detections divided by total event detections) varying between 0.2 and 0.4 and the recall ratio (correct detections divided by total actual events) never climbing higher than 0.8. The authors also investigated a maximum likelihood estimation (MLE)-based approach in which live measurements were matched to one of two histograms respectively representing an ’event’ state and a ’silent’ state. These histograms were created for each link during an offline training phase. Unfortunately, this approach did not solve the issue of false positives and a slightly higher recall ratio of 0.9 was contrasted with a precision that barely reached above 0.2.

Another early DFL-based detection system was proposed by Lee et al. [43] which made use of only a single pair of IEEE 802.15.4 compliant nodes. The general principle behind their approach was the fact that histograms of the RSS fluctuations (defined as the difference between the current and the previous RSS measurement) were much narrower if there was no human movement near the communication link. The authors devised a system that counted the number of small RSS-fluctuations between −1 and 1 over a window of *N* packets. If the percentage of these types of fluctuations fell below a predefined threshold, movement would be detected. This system was experimentally validated in three different meeting room environments with sizes of approximately 6 m by 4 m. Measurements were performed during a period of 20 min, with the transmitting node broadcasting a packet every 250 ms. At 10 predefined moments during this time period, a human would enter the environment and continuously walk between the two nodes for 30 s. In all three environments, the system managed to successfully detect each human walk within 15 s. In two of the three meeting rooms, occasional false positives were detected as well, however. By tuning the value of parameter *N*, the amount of false positives within a 20-min experiment could be reduced to two for both environments.

#### 2.1.2. RASID

The detection-related experiments discussed in the previous section demonstrated how non-complex setups could still obtain reasonably accurate results, but the proposed systems could hardly be considered robust. In 2012, Kosba et al., created RASID, one of the first full-fledged robust DFL systems solely focused on detection [44]. This system was capable of accurately detecting the presence of (human) motion in both a single floor office environment of approximately 186 m^2^ and a two-floor home building with each floor having a size of approximately 139 m^2^, while only making use of RSS measurements from a maximum of 12 different communication links.

RASID consisted of both an offline training phase and an online monitoring phase and made use of the moving RSS variance over a sliding window as its main feature value for event detection. During this offline phase, an initial silence profile was constructed based on calibration measurements when the environment did not contain any human motion. For each link, a density function of observed variances was estimated through the use of a non-parametric kernel density estimation technique. Based on a predefined significance parameter α, an upper bound was then defined whose value was equal to the 100(1−α)-th percentile of the cumulative distribution function of the estimated density. Measuring variances above this upper bound during the online phase would signify the detection of an anomaly and lead to the declaration of a global alarm. Additionally, the ratio between the measured variance and the upper bound was calculated and defined as an ‘anomaly score’. These scores would be used to determine if the silence profile could be updated with current measurements. Additionally, anomaly scores from multiple links were combined to make a final decision whether motion was detected or not. Interestingly, RASID also offered a very rudimentary form of location estimation based on the anomaly scores of the different links.

The performance of RASID was evaluated by installing four APs (Cisco Aironet 1130 AG series access points) and three MPs (laptops containing D-Link AitPlus G+ DWL-650+ Wireless NICs) in the two environments mentioned previously. An important metric that was used was the F-measure. It is defined as the harmonic mean of the precision and the recall and is commonly used to measure the accuracy of binary classification algorithms. RASID was compared to the moving average, moving variance and MLE-based approaches discussed in the previous section and generally outperformed these techniques, with F-measures of respectively 0.96 and 0.93 for the office and home environments. Only the MLE-based obtained a slightly better result. However, a second experiment in which the online monitoring phase occurred two weeks after the offline construction of the initial silence profile did make the advantages of the robustness of the RASID system quite clear. F-measures of 0.94 and 0.92 were obtained—only slightly lower than in the first experiment—while the MLE-approach suffered from large amounts of false positives and only obtained F-measures of 0.60 and 0.65. These results clearly demonstrated that RASID was an important step forward within DFL as one of the first truly robust RF-based device-free detection systems.

#### 2.1.3. FIMD

Soon after RASID, another robust DFL-based detection system called FIMD (Fine-grained device-free motion detection) was proposed by Xiao et al. [45]. Its detection algorithm did not make use of RSS values, but instead used CSI obtained from commercially available IEEE 802.11n Wi-Fi chips. CSI provides a much clearer overview of the manner in which a communication channel behaves by containing both amplitude and phase measurements for every single subcarrier in an orthogonal frequency division multiplexing (OFDM) communication system. The authors initially considered the use of CSI over RSS for a detection system to be advantageous for three different reasons. First of all, it was considered to be much more resistant to narrowband interference from other 2.4 GHz signals due to the fact that it describes channel characteristics based on a large, frequency-diverse group of subcarriers. Second, CSI values were assumed to be much more stable in an entirely static environment that does not contain any motion. Finally, CSI was considered to be entirely independent of automatic power adjustments that can occur in off-the-shelf commercially available access points.

The main feature value used for motion detection within FIMD was derived from the CSI measurements as follows. Sliding windows of a certain fixed size iterated over the raw CSIs and for each window, a square matrix **C** was constructed which contained the correlation ratios between all possible combinations of CSI measurements within that window. Eigenvectors were then determined for this matrix **C** for which the corresponding eigenvalues were maximized (after normalization over the sliding window size). Generally, the maximum and second maximum eigenvalues were used as the feature values **V**. The general idea behind the use of these values lay in the fact that higher correlation ratios between the raw CSI measurements within a single-window were assumed to correspond to static environments, whereas a sudden decrease could potentially indicate motion.

An evaluation of the system was performed in two different test environments: a 7 m by 11 m laboratory and a narrow 32.5 m by 1.5 m corridor environment. In both cases, a TL-WR941ND router with three antennas was used as AP and an HP laptop containing an iwl5300 IEEE 802.11n NIC was used as MP. Only the first antenna of the NIC was used, with the authors noting that the use of multiple antennas would be left for future work. For both environments two test sets were collected, one of which was static and one of which was created when an individual was continuously walking within the test environment. Applying the FIMD-algorithm to the collected data showed that detection rates greater than 90% could be obtained in the lab environment. This did require a selection of parameters which also led to false-positive rates of 14% or higher, however. If parameters were optimized in order to reduce false-positive rates to below 1%, the detection rates would drop to just higher than 70%. The trade-off was less harsh in the corridor environment, as detection rates above 90% were measured when the corresponding false-positive rate was approximately 9%. A direct comparison with RASID was also performed, which indicated that FIMD slightly outperformed the purely RSS-based system despite not requiring an offline training phase. While this increase in accuracy was quite minor, this study nevertheless managed to show that the use of CSI-based features was feasible and could lead to improved results.

### 2.2. Crowd Estimation

**Figure 4:**
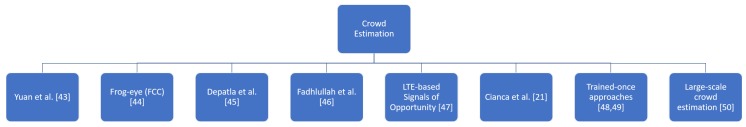
A schematic overview of the main crowd estimation approaches discussed in this survey.

In this subsection, we will discuss device-free detection approaches in which the focus lies on estimating the amount of targets present in an environment. A schematic overview of the most important crowd estimation techniques is provided in Figure 4.

In 2013, one of the first studies regarding the potential use of WSNs to estimate crowd densities was detailed by Yuan et al. in [46]. They installed 16 TelosB nodes operating in the 2.4 GHz frequency band in a grid-like fashion within an empty room measuring 18 m by 18 m. These nodes regularly broadcasted packets to one another which contained RSS measurements with which previously transmitted packets had been received. All these RSS values were captured by a special sink node which was connected to a laptop on which the crowd estimation algorithm could be run. By making use of a modular, training-based approach in which the densities of subregions were taken into account, crowd density levels within the environment could be estimated. These levels were defined as low (0–3 targets), medium (4–10 targets) and high (more than 10 targets). On average, estimation accuracies of 94% and 86% were obtained when the experimental crowd was respectively static or moving. Additionally, a large-scale simulation was performed in a 100 m by 100 m environment containing 400 nodes. The results of this simulation (an estimation accuracy of 83%) seemed to strengthen the feasibility of the concept.

One of the first full-fledged RF-based crowd counting systems was proposed by Xi et al. in 2014 [47]. Their technique was called FCC (device-Free Crowd Counting) and made use of CSI data from a limited amount of 2.4 GHz 802.11n communication links. The main idea behind their technique was to make use of a specific metric called PEM (Percentage of nonzero Elements), which could be derived from the raw CSI values of a single link. The derivation of this metric occurred according to a three-step process. First, measured amplitude CSI values were transformed into a two-dimensional matrix. During the second step, the matrix was dilated. Finally, the percentage of elements within this dilated matrix whose value was not equal to zero was calculated. Based on a few training measurements that were taken when the environment contained different amounts of human individuals, a grey verhulst, odel could be constructed which described the relationship between PEM and the number of targets present in the environment. The resulting crowd estimates from each link were then combined through the use of a weighted average.

In order for this technique to be scaleable towards large environments in which different links could provide crowd estimates for different subregions, the authors also investigated a method to prevent too much overlap between these subregions. This method consisted of attempting to filter out the majority of information not related to (near) line-of-sight paths from the CSI measurements. CSI provides both the amplitude and phase of each subcarrier of an OFDM Wi-Fi signal and as a result can be seen as a representation of the frequency domain channel response. Therefore, a time-domain channel response could be obtained by performing an inverse fast Fourier transform (IFFT). A truncation window was then used to remove most non-line-of-sight paths, after which an FFT provided the authors with filtered CSI values that could be used in the main system.

Extensive experiments were performed in a multitude of both indoor and outdoor environments in which the impact of several model parameters (e.g., matrix dilation coefficient), crowd properties (e.g., crowd speed) and system setup properties (e.g., Tx–Rx distances) were analyzed. The sizes of the crowds in these test setups went up to 30 human targets. Generally, it was concluded that FCC performed well outdoors and exceptionally well indoors, with approximately 70% of estimation errors being off by less than two targets in the outdoor case. For the indoor case, this percentage was equal to 98%. The authors hypothesized that the more obvious multipath in indoor environments effects were the underlying cause of this difference. Given these highly impressive results, FCC can justifiably be seen as an important milestone within the evolution of the field.

Another impressive crowd counting framework was proposed by Depatla et al. [48]. The authors described a methodology which enabled accurate crowd estimations of up to nine human individuals in both indoor and outdoor environments, while only using RSS measurements from a single Wi-Fi one-way communication link. In this proposed methodology, the influence of a single (walking) individual on the measured RSS values of communication links was considered to consist of two separate aspects: blocking of the line-of-sight and contribution to multipath effects. This first aspect was mathematically characterized in a probabilistic manner through the development of a simple motion model. For the second aspect, the scattering caused by human targets was mathematically characterized as well through probabilistic analysis of multipath fading as a function of the number of targets. Combining these two aspects led to a full-fledged model that described a unique RSS probability distribution based on the number of targets.

When the system was active, the actual RSS distribution based on measurements was compared to the distributions that resulted from this model. The modeled distribution that was closest to the measured distribution according to the Kullback–Leibler divergence metric was selected. The parameter N describing the number of targets that led to this modeled distribution was then considered to be the final crowd estimate of the system. Interestingly, the authors also investigated a shorter and less complex methodology which only took the first aspect into account and could be used for setups in which the nodes had directional antennas.

Experiments were performed in both a 7 m by 10 m outdoor and a 4.4 m by 7.5 m indoor environment. Generally, errors of two targets or less were achieved in respectively 96% and 63% of cases for the outdoor and indoor cases when using omnidirectional antennas. For the system that made use of directional antennas, this percentage was equal to 100% in both environments. It should be stressed that these results were obtained with a system that only made use of RSS measurements from a single link. The authors themselves noted that this held much promise for further extensions within this field of research.

Another interesting study was performed by Fadhlullah and Ismaili in [49]. They analyzed the impact of a crowd on the RSS values that were obtained from communicating Zigbee nodes in an environment measuring 20 m by 5 m. Interestingly, their setup consisted of a single, static main node that was installed on one side of the environment and three semi-mobile nodes that were carried by three human individuals standing on the other side of the environment. Communication occurred only between the static node and each semi-mobile node, which meant that there was only data from three links that could be used to estimate the crowd density. The crowd whose density we wished to analyze was present in the middle of the environment. The statistical approach that was used led to accuracies of respectively 75.00% and 70.83% when trying to estimate low (five targets) and medium (10 or 15 targets) crowd level densities. Furthermore, their research led the authors to conclude that the impact of a crowd on the measured RSS values of 2.4 GHz communication did not statistically differ depending on whether the individuals in the crowd were moving or were entirely static.

Several of the systems discussed in the previous paragraphs managed to obtain impressively accurate crowd estimation results with setups containing extremely low amounts of communication nodes. In [50], Di Domenico et al. took this even further by proposing a passive crowd estimation approach that made use of already present LTE ‘signals of opportunity’. They installed a single SDR platform in a ground floor meeting room measuring 5 m by 9 m which was capable of receiving the Pilot signals transmitted by a standard LTE eNodeB transmitter at a distance of approximately 560 m. The captured signals were then analyzed by calculating a multitude of variation-related features of the reference signal received power (RSRP) for each channel. The general idea behind this approach was the assumption that the measured amplitude variations were correlated with the amount of people present within the room in which the receiver was installed. Experiments performed in the previously mentioned environment led to classification accuracies between 76% and 92% depending on the location of the SDR equipment. A class could represent the presence of either 0, 1, 2–3 or 4–5 individuals. While the authors themselves explicitly indicate that these results were preliminary and that they do not claim accuracy and precision in every type of environment, they were nevertheless highly impressive. The use of signals of opportunity within DFL is a highly interesting future direction for this field of research—especially considering the increasing emergence of IoT networks in city environments. We will discuss this in more detail in Section 5.

Another interesting set of passive crowd estimation experiments was performed by Cianca et al. [22]. While the authors of this paper focused primarily on providing an overview of RF-based passive sensing in general, they also investigated and compared the accuracies of an RSS-based and a CSI-based methodology. In particular, they stressed an important difference between the use of RSS and CSI: frequency selectivity. RSS measurements of an OFDM signal can be regarded as representative of the average attenuation over all subcarriers. If the communication channel is not very frequency selective, a CSI measurement will not show much variation between the different subcarriers and will therefore not provide much more information compared to an RSS measurement.

In their experiments, the authors made use of a single commercially available IEEE802.11b Wi-Fi AP and captured its periodically transmitted beacon messages using an SDR platform. For both their RSS and CSI-based approaches, two different features were derived from their respective measurements within a sliding window of a certain size and were then used to train a Linear Discriminant Classifier. The selected features for RSS were the standard deviation and the normalized standard deviation while for CSI the spectral distance mean and spectral distance standard deviation were used. Spectral distance in this context was defined as the mean normalized Euclidean distance between the amplitude spectra of two subsequent beacon packets. It represented the manner in which the frequency selectivity of the communication channel varied in time over consecutive beacons.

The experiments took place in two different environments in which up to five targets could be present. The first environment was a 5 m by 6 m office room in which movement by the targets was constrained to locations close to the transmitter or receiver due to a large amount of furniture that was present. The second environment was a 5 m by 9 m meeting room which contained a large amount of metal chairs but had much less movement restrictions. The resulting crowd estimation accuracies for the office environment were equal to respectively 72% and 70% for the CSI and RSS-based approaches. A much larger difference was observed for the meeting room environment, with a CSI-based estimation accuracy of 63% and an RSS-based accuracy of 57%. These results were in line with expectations, with the experiment showing that RSS-based approaches can still obtain impressive results in small-scale environments where targets are forced to move in the vicinity of the transmitter and/or receiver equipment and will therefore primarily influence the communication signals due to the direct blocking of the LoS. In order to obtain reasonably good results in both environments, however, the CSI-based spectral distance approach was required. Additionally, it was remarked that the amount of features that can be derived from RSS measurements is rather limited, while there are still many more possibilities to increase the selected feature set when using CSI. In conclusion, the authors stated that the performed experiments contributed to a more thorough understanding of the impact of the selected features and the specific environment. However, they also indicated a lack of more theoretical models as a current weak point of the state-of-the-art of RF-based passive sensing.

A common issue in many of the experimental crowd estimation systems discussed so far is their requirement of collecting training data in the exact same environment in which the live system will operate. In [51], Di Dimenico et al. took the first steps towards a solution for this problem with the development of a CSI-based, trained-once crowd counting method. The underlying idea of their approach was the intuitive assumption that an increasing amount of moving targets in the environment will lead to larger multipath variations due to them acting as scatterers. As a result, the variation of CSI measurements overtime was assumed to depend primarily on the number of moving targets, rather than the specific characteristics of the static environment in which the measurements took place. In the proposed technique, a pair of variation-related features was derived from CSI measurements collected in a single training environment and used to train a linear discriminant classifier. Next, an evaluation phase took place in two different environments. The environments in which experiments took place were all meeting rooms of different sizes (30 m^2^, 45 m^2^ and 75 m^2^) containing up to seven human individuals. The medium-sized room was used for training. Each environment contained a single Wi-Fi access point/Wi-Fi receiver pair installed on opposite sides. The AP transmitted over two antennas while the receiver used three, providing the experimenters with measurements from six different communication channels. The results showed that in more than 91% (small room) or 81% (large room) of cases, the estimation was off by two targets or less. These were promising results, clearly indicating that an important first step had been taken regarding the issue of training in crowd estimation systems. Interestingly, it should be noted that the final part of the paper describing this approach contains a brief overview of other existing crowd estimation techniques at the time, many of which we have also discussed ourselves in the previous paragraphs of this section.

Di Dimenico et al. continued their research regarding this approach in [52]. In their improved method, they did not directly calculate variation-based features based on the raw CSI data. Instead, they used the CSI measurements to calculate a mean Doppler spectrum for each communication channel and derive classification features from these spectra. Additionally, they used a naive Bayesian classifier to assign a set of features to a crowd estimation class, rather than the previously used linear discriminant classifier. In highly similar setups in the same environments, their new technique managed to obtain classification accuracies of respectively 73% and 63% in the small and large meeting rooms, with data from the medium-sized meeting room having been used for training. This indicated relatively small but clear improvements when compared to the previous technique.

Another interesting CSI-based crowd counting method called WiFree was developed by Zou et al. in [53]. They strongly focused on the opportunities for passive sensing in smart buildings offered by an ever-growing IoT. The first step of their approach, therefore, consisted of the development of an OpenWrt-based [54] IoT platform for commercially available WiFi-routers. The installation of this firmware enabled these hardware devices to also be capable of acting as a receiver themselves (instead of using laptop NIC cards or full-fledged SDRs) and allowed the researchers to obtain an increased amount of CSI-data. Next, their detection and counting approach itself was based on the use of a transfer kernel learning-approach. Experiments involving three different environments that could contain respectively up to 4, 7 and 11 individuals managed to achieve a 99.1% occupancy detection accuracy and a crowd counting accuracy of 92.8%.

In many of the research papers, we have discussed in the entire crowd estimation subsection thus far, the potential for using crowd estimation techniques to enhance crowd safety is regularly mentioned [46,49,50]. While this would indeed be a highly interesting application domain, the experiments that have been performed thus far took place in relatively limited environments where there were—at most—no more than a few tens of targets present. In order to investigate whether a sensorless sensing approach would still be useful to estimate the crowd density at actual large events, we set up an experiment ourselves as described in [55]. In this study, we installed 46 battery-powered nodes around the edges of a single stage at a large music festival. This highly realistic test environment had a size of approximately 45 m by 39 m and could contain thousands of human individuals while the festival was ongoing. Each node was placed at a height of around 1 m and contained both an 868 MHz and a 433 MHz self-developed EZR-USB transceiver. Communication between the transceivers in different nodes occurred by using the DASH7 alliance protocol (D7AP) [56], while the two transceiver modules within the same node did not interact with each other in any way.

We hypothesized that the average attenuation experienced by the communication links within our RF-network could be indicative of the true crowd size. Attenuation in this context was defined as the RSS difference between an online link measurement and an offline calibration measurement which was performed when the environment was (largely) unoccupied. In order to properly investigate this, it was necessary to have access to some form of ground truth data which could provide us with the actual number of human individuals that were present in the environment. Due to the fact that our setup was installed in a real, uncontrolled environment in which a commercial event was taking place, it was not possible for us to control the crowd and order exact amounts of human individuals to enter or exit the environment. Unfortunately, no other crowd estimation systems were present during our experiments. Instead, we made use of a large collection of very low-quality camera images and had them manually analyzed by a large team of volunteers. Each image was classified into one of six categories numbered from 0 to 5, with category 0 corresponding to an environment that was nearly empty, while category 5 indicated an environment that seemed to be filled to the brim with people. These classifications acted as our ‘visual validation’ data. Next, we trained a probabilistic neural network (PNN) using a set of 433 MHz + 868 MHz average attenuation values and their corresponding categorizations and evaluated its classification accuracy using the rest of the data. This step was repeated 50 times according to a Monte Carlo cross-validation approach and the results were averaged. These final results indicated over 90% of all estimations by a trained PNN were at most one category removed from the category that was determined by our visual validation data. Furthermore, there were some indications that the less-than-stellar visual validation system we utilized was (partially) responsible for the inaccuracies that did occur. As a result, we concluded that—although it should be kept in mind that this study was still preliminary—it was feasible to make use of an RF-based WSN approach for crowd estimation in realistic, large-scale environments.

An overview of the most important detection techniques which we discussed in this section is provided in Table 1.

## 3. Tracking

In this section, we will discuss the evolution of tracking-focused DFL techniques. Depending on its specific methodology, each technique is categorized as being based on either radio tomographic imaging, a non-imaging measurement model or passive fingerprinting.

### 3.1. Radio Tomographic Imaging

Radio tomographic imaging is a device-free localization technique which utilizes the impact of a non-static entity on the RSS values of RF communication links in order to determine the position of this entity. RTI was first proposed by Wilson et al. [40] and since then the technique has attracted an increasing amount of research interest. Its use of low-cost hardware, through-the-wall imaging capabilities and low computational requirements for real-time applications are major advantages. Many variants of the basic algorithm do exist, ranging from variance-based RTI (VRTI) [57] which does not require calibration measurements to multi-tracking systems capable of tracking up to four people simultaneously [58].

**Figure 5:**
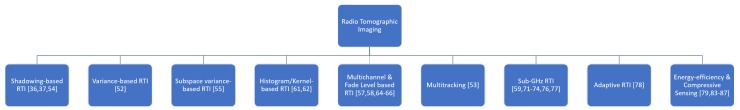
A schematic overview of the main radio tomographic imaging approaches discussed in this survey.

In this subsection, we provide an overview of the evolution that research into RTI has undergone over the past decade and discuss several key RTI variants and improvements. A schematic summary of the main approaches we discuss can be found in Figure 5.

#### 3.1.1. Shadowing-Based RTI, Variance-Based RTI and Subspace Variance-Based RTI

The original RTI algorithm proposed by Wilson and Patwari [40,41] is called shadowing-based radio tomographic imaging. RF nodes in an environment communicate with each other through transmitting radio signals. Both static and non-static objects in the environment will influence the radiated power of these signals through absorption, reflection, diffraction or scattering. By calculating the differences between RSS-measurements performed while the system is active and offline calibration measurements performed when the environment did not contain any non-static objects, one can eliminate the impact of the static elements. In RTI, the collection of all current RSS-difference measurements is represented by a vector *y*, which has a size M that is equal to the total amount of communication links within the RF-network. Vector *y* can be described by the following equation:

(1) y=Wx+n.

The goal of the RTI algorithm is to approximate the image vector *x*. Each element of this vector corresponds to a location in a rasterized representation of the environment and indicates the average attenuation that a link will experience when its direct line-of-sight crosses that location. It is assumed that locations with a high amount of average attenuation are likely to contain the targets we wish to estimate the locations of. *W* is an *M* × *N* weighting matrix, with *M* equal to the total amount of communication links and *N* being the amount of ‘pixels’ or elements the image vector contains. Each column of *W*, therefore, represents a single pixel and each row describes the weighting of each pixel for a single link. An ellipse is defined for each link which determines the locations that are considered to be able to have an impact on that link. The nodes which comprise the communication link are the foci of this ellipse. If the location represented by a certain pixel falls outside of the ellipse, the location is assumed to not have any influence on that link and the corresponding weighting value will be set to zero. It should be noted that this elliptical model is a severe simplification of the manner in which RF-waves propagate throughout an environment. Furthermore, RTI considers the impact of multipath effects (e.g., fading gain) to be part of the noise. While this assumption greatly lowers the complexity of the algorithm, it nevertheless means that ‘noise’ will increase if there is more multipath. Therefore, the accuracy of RTI will suffer in complex environments.

The value of each element of *W* is given by the following equation:

(2) $$W_{ij}=\left\{\begin{array}{c} 1/\sqrt{(}d_{i})\;\;\mathrm{if}\;d_{ij}\left(1\right)+d_{ij}\left(2\right)<d_{i}+\lambda ,\\ 0\;\mathrm{otherwise}, \end{array}\right.$$

with *d* being the distance between the nodes comprising a link, $d_{ij}\left(1\right)$ and $d_{ij}\left(2\right)$ being the distances between pixel *j* and the node locations and λ being an excess path length parameter (which uses pixels as units) responsible for the width of the ellipse.

Equation (1) is an ill-posed, inverse problem. A single, unique solution does not exist and needs to be approximated. In basic shadowing-based RTI, a maximum a posteriori (MAP) approximation is used. When applied to (1), this leads to the following formula:

(3) $$x_{MAP}={\left(W^{T}W+C_{x}^{-1}\sigma_{N}^{2}\right)}^{-1}W^{T}y,$$

where $C_{x}$ is an a priori covariance matrix which assumes *x* is a zero-mean Gaussian random field and $\sigma_{N^{2}}$ represents noise variance. This term is used for regularization. Were it not present, Equation (3) would be a simple least-squares solution. This would only be valid if the matrix *W* were full rank (to ensure that $W^{T}W$ is invertible), which is (almost) never the case in RTI. Furthermore, a least-squares solution amplifies the noise so strongly that the result would become essentially meaningless.

Wilson et al. investigated potential alternative regularization techniques for RTI in [59]. They compared the performance of three techniques: Tikhonov regularization truncated singular value decomposition (TSVD) and total variation. The authors concluded that Tikhonov regularization is very appealing for RTI due to its flexibility regarding the incorporation of desired characteristics in the resulting images. Furthermore, its low computational complexity made it a perfect fit for real-time RTI systems. TSVD, on the other hand, does not require (or even allow) prior information about the solution. The resulting images with this regularization technique were considered too noisy and lacked the contrast needed for accurate localization. Finally, total variation RTI images showed a large amount of contrast and a low level of noise, but the technique required two regularization parameters and was computationally expensive.

In practice, many RTI implementations tend to either use Tikhonov regularization [57,60,61] or a least squares solution in combination with a covariance matrix [62,63,64,65] similar to the MAP approximation in Equation (3).

An important advantage of Equation (3) lies in the fact that with the exception of vector *y*, all other parameters can be determined while the system is offline. This provides important benefits regarding the required processing time for real-time applications and allows for the following simplified equation:

(4) $$x_{MAP}=\Pi y,$$

with Π being a linear transformation matrix which was calculated before the system was active.

Wilson and Patwari showed the potential of this technique for the first time with an experiment described in the initial RTI tech report [40]. The 28 2.4 GHz RF nodes were placed in a square fashion in a large indoor environment. The area demarcated by the nodes measured 4.27 m by 4.27 m, with the nodes on each side of the square spaced 0.61 m apart. Calibration measurements were performed when no one was present within this square, after which test subjects were asked to walk around in the environment. Resulting image vectors clearly indicated sources of attenuation in locations where people were present. In [41], the authors described and analyzed the basic RTI technique more thoroughly and performed a new experiment in an outdoor environment which had a size of 6.40 m by 6.40 m. As was the case for the previous experiment, 28 nodes were placed around the edges. Interestingly, this environment was not entirely open and contained two trees. Just as in the previous experiment, the results showed an increased attenuation in locations where a target was present as well.

**Figure 6:**
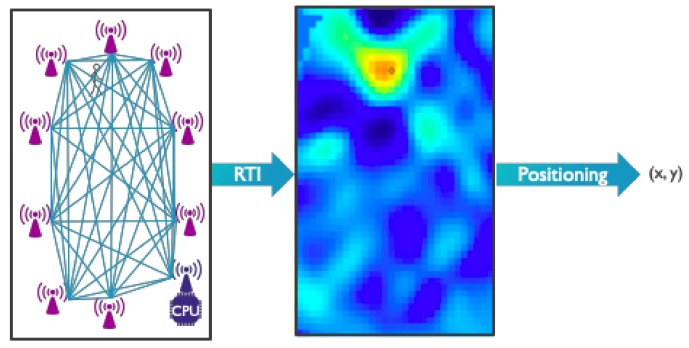
Schematic overview of the radio tomographic imaging (RTI) process.

It is important to note that the RTI algorithm in and by itself only creates an attenuation image and does not directly provide target location estimates. As indicated in Figure 6, an additional positioning step is required. This can be as simple as using the coordinates of the pixel with the highest amount of attenuation [57], or it can encompass the use of complex machine vision techniques [58]. Furthermore, when tracking moving targets the estimates from the positioning step can be used as input to a motion filter (e.g., a Kalman filter) which determines the final location estimate.

A major disadvantage of shadowing-based RTI as described in the previous paragraphs is the fact that calibration measurements need to be performed when the environment is static. This makes the technique not suitable for emergency, police or military applications where the targets are already inside the environment in which they need to be located. To tackle this issue, Wilson et al. [57] developed Variance-based RTI which eliminates this requirement by making use of the variances of the RSS values of each link. A moving object in an RF multipath environment will influence the amplitude or phase of one or more multipath components over time. This will cause the phasor sum of all components at the receiver to change and therefore lead to an increase in RSS variance. In VRTI, vector *y* (from Equations (1), (3) and (4)) contains the continuously updating windowed variances of the communication links, rather than the RSS-difference values. Variance was experimentally shown by Wilson et al. to be a much more stable indicator of motion within the environment. An important disadvantage of VRTI, however, is its inability to locate an entity that has become entirely stationary. When designing a full-fledged RTI system, this needs to be taken into account (e.g., by incorporating this aspect into the motion model).

Subspace variance-based RTI (SubVRT) is a variant of VRTI developed by Zhao et al. in [60]. This approach takes into account the fact that motion within an environment does not necessarily have to be caused by the entities we wish to track, but can also be an intrinsic part of the environment (e.g., moving machinery parts). A subspace decomposition method closely related to principal component analysis is used to create projection matrices for the intrinsic and extrinsic subspace. Using these matrices to project the variance vector *y* that is used in regular VRTI to extrinsic subspace causes us to obtain an extrinsic signal component vector $\tilde{y}$. Replacing *y* with $\tilde{y}$ in Equation (3) leads to the subVRT solution which does not contain any intrinsic components. A downside of this approach is the fact that in order to obtain separate projection matrices for intrinsic and extrinsic motion, calibration measurements are necessary. Therefore, an important advantage of VRTI over shadowing-based RTI is nullified. It should additionally be noted that subVRT is only feasible for use in environments in which the intrinsic motion can be easily modeled based on a single set of calibration measurements. If the intrinsic motion is much more unpredictable—as is the case in long-term outdoor setups—an entirely different approach is required. These types of setups were only first analyzed in 2016 by Alippi et al. [65].

#### 3.1.2. Histogram- and Kernel-Based RTI

As stated earlier, regular VRTI-based systems are not capable of estimating the location of stationary targets. In response to this shortcoming, Zhao et al. developed histogram distance-based radio tomographic imaging (HD-RTI) [66,67]. In this RTI variant, each communication link is characterized by two RSS histograms: a short-term histogram (STH) and a long-term histogram (LTH). The general idea behind the technique is that the STH and LTH will only differ in a significant manner if there are targets present near the communication link. The variance vector y that is used in VRTI is reinterpreted as a histogram distance-vector, in which each element represents the difference between the STH and LTH for the corresponding link. Many different techniques exist to compute the distance between two histograms. Zhao et al. examined two in particular: Kullback-Leibler divergence (KLD) and kernel distance. Kernel distance was found to provide more accurate localization results and as a result, most HD-RTI systems are called kernel distance-based RTI (KRTI). Calculating kernel distance requires the use of a kernel function. Both Gaussian and Epanechnikov kernel functions were tested and they were shown to achieve similar performance.

The performance of HD-RTI/KRTI was extensively researched. A total of five datasets from different experiments were used in [67] to evaluate the technique and compare its accuracy to other tracking approaches: VRTI, subVRTI and SMC (a non-imaging technique which will be discussed later). This data collection was comprised of two datasets re-used from the earlier VRTI research of Wilson et al. described in [57], two datasets created based on new measurements performed in the same environment and one dataset based on a new experiment in a 12 m by 5 m bookstore where 34 transceivers were deployed. The tracking of both static and moving targets was investigated, as well as the impact of intrinsic motion and several model parameters. The results nearly consistently indicated the superior performance of KRTI for a variety of different conditions and environments when compared to the other algorithms at the time.

#### 3.1.3. Multichannel and Fade Level Based RTI

In all of the RTI variants discussed so far, communication between the nodes occurred on a single frequency channel of a 2.4 GHz communication protocol. Kaltiokallio et al. proposed channel diversity RTI (cdRTI) [68] which attempts to improve the accuracy of basic shadowing-based RTI by making use of multiple frequency channels. The general idea is to select the communication channels for each link that are most suitable for performing RTI in a specific environment and combine the measurements performed on these channels. This selection occurs based on the concept of link fade level. The fade level of a communication link describes the amount of destructive multipath interference that this link experiences when the environment is static. It is quantified as the difference between the mean RSS value that is measured when the environment is static and the ‘expected’ RSS value as determined by the log-distance path loss model. Links in deep fade experience a large amount of destructive multipath interference and are more likely to show a higher RSS variance when a target is near. The opposite phenomenon can be observed in links that are in antifade. They experience a high level of constructive multipath interference and show a smaller variance when a target is in the vicinity. Only when the target is standing directly in the line-of-sight will there be a noticeable impact on the link in the form of a high amount of RSS attenuation. Many links are neither in deep fade nor in antifade, but lie somewhere in between. Because links in antifade will primarily cause RSS attenuation when a target stands directly in its line-of-sight, they are preferable for techniques that make use of attenuation such as shadowing-based RTI. A strongly negative fade level indicates a link in deep fade while a strongly positive fade level indicates a link in antifade. The channels are ranked and the *n* channels with the highest fade level are selected. Given the fact that fade levels in this technique are only used in a relative manner to rank different channels, they can be substituted by the power-normalized RSS values (changes in large-scale path loss and shadowing loss caused by the slightly different center frequencies of different channels are very minor and are therefore not considered to be relevant).

The actual RTI algorithm is performed during the next step, while the system is active and live measurements are being performed. As is the case in most RTI variants, the goal is to approximate an image vector x and Equations (1) and (3) are applied here as well. The only difference with shadowing-based RTI is that each element of vector y is determined by calculating the mean of the measured RSS attenuations on the n selected channels. Experiments to investigate the capabilities of this channel diversity approach were performed in two different environments: an open indoor environment where there was a clear line-of-sight between all nodes and a complex through-the-wall lounge room containing many static objects. The 30 IEEE 802.15.4-compliant RF nodes were placed in these environments and they communicated on five different channels. Furthermore, during the experiments a nearby network that communicated on one of these channels was present, thereby causing interference. For both environments, the goal was to estimate the location of a stationary test subject who was standing in certain predefined locations. In the open environment, localization errors of 0.10 m were obtained when the method selected the four most suitable channels. Additionally, a localization error of 0.96 m was obtained in the lounge environment with the method selecting the three most suitable channels. This meant that an attenuation-based RTI method had been developed that was capable of locating targets in through-the-wall situations with sub-meter accuracy.

Fade level RTI (flRTI) is another RTI variant that combines the concepts of multiple channels and link fade levels in [62]. One of the most important novelties of this technique was the fact that the parameter λ in the weight model shown in Equation (2) was no longer static. This parameter plays an important role in defining the size of the ellipse that determines which parts of the environment are able to influence a link. Each frequency channel on each communication link now had two separate values for λ based on fade level: one for when an RSS increase was measured and one for when a decrease was observed. Extensive training data was used to compute the parameters for the fade level-based spatial weight model that described λ as a function of the fade level. This technique also used training data to construct a fade level-based measurement model that was capable of directly transforming each measured RSS attenuation into a value that described the probability of a target is present in the area delineated by the corresponding ellipse. Furthermore, flRTI did not apply a channel selection method like cdRTI. Instead, RSS measurements of a frequency channel were transformed into probabilities and put into one of two-channel vectors $y_{c}^{-}$ or $y_{c}^{+}$ depending on whether attenuation or signal increase was measured. This was done for all channels that were used for communication in the deployed RTI network. A concatenation of both $y_{c}^{-}$ and $y_{c}^{+}$ led to the complete measurement vector $y_{c}$ for a specific channel and a subsequent concatenation of all channel vectors led to the final measurement vector *y*. This meant that, for an RTI setup with *L* communication links and *C* number of channels, the measurement vector would have a size of 2LCx1. Correspondingly, the size of the weighting matrix also changed to 2LCxN, with *N* being the total amount of pixels in the discrete representation of the environment.

Three different experiments were set up to investigate the performance of flRTI in comparison to cdRTI. In all cases, 30–33 RF nodes that communicated on 4 to 5 different frequency channels were set up in an environment. The flRTI was shown to consistently outperform cdRTI in both an open environment, an apartment, and a complex through-the-wall environment. One noticeable disadvantage, however, was the fact that both the offline computation of the main linear transformation matrix Π (see Equation (4)) and the online approximation of the image vector *x* required much more computing time. These results were unsurprising, given the increased sizes of the measurement vector and the weighting matrix as described in the previous paragraph. Nevertheless, these experiments showed the validity of flRTI and definitively proved that a model based on RSS attenuation is certainly viable for complex through-the-wall scenarios.

All of the fade level-related RTI research we have discussed so far indicates that communication links in antifade are preferable to deepfade links for attenuation based techniques. This is even the whole point of cdRTI, where channel selection based on fade level achieves superior accuracy-related results. A highly interesting implementation of flRTI was performed in [63] that aimed to increase the fade levels of the communication links in the RF sensor network through the use of servo-nodes. These nodes consisted of the RF sensor itself, a rigid cardboard circle to which it was attached and a rotating servo motor whose winch was attached to the cardboard. The servo motors were capable of making small adjustments to the positions of the nodes which could have a large impact on the measured RSS values due to small-scale fading effects. This concept was used to have each servo-node optimize its own position in order to maximize the sum of all RSS values measured in the environment during the calibration measurement. This increases the average link fade level in the network and therefore improves the tracking accuracy. Experimental implementations of servo-nodes in three complex test environments (56 m^2^ apartment, 54 m^2^ laboratory and 100 m^2^ office space) led to reductions in flRTI localization errors between 30% and 37% when compared to a network that used static nodes. This demonstrated the large impact that even small changes in node placement can have. Additionally, these results solidified once more the value that the concept of link fade level has in RTI.

#### 3.1.4. Multitracking

Nearly all of the RTI variants we have discussed so far focused primarily on the localization and tracking of a single target. Furthermore, the assumption was made that the number of targets was known. For many real-life applications, a multitracking system must be able to correctly estimate the number of targets that need to be tracked. In other words, it must be able to perform both detection and tracking.

Bocca et al. proposed a multitracking method for RTI in [58] that was capable of tracking up to 4 targets simultaneously in cluttered indoor environments. They implemented a multichannel RTI system that attached a weight to each measured RSS attenuation based on relative fade level. Relative fade level for each link–channel pair was calculated as the difference between the measured path gain for that channel and the lowest measured path gain on that link. The resulting image vectors were regarded as frames of a video on which machine vision techniques could be applied. If the path of two or more moving targets intersected, blobs in the corresponding RTI images would merge into a single one and split again after some time. The system automatically detected such occurrences by constantly checking the distance between targets and dynamically updated certain parameters to take this special situation into account. Experiments were performed in three different environments: an open 70 m^2^ indoor environment in which 30 nodes were installed, a one-bedroom apartment of size 58 m^2^ in which 33 nodes were installed and an office environment of size 67 m^2^ in which 32 nodes were installed. The nodes communicated on five different frequency channels for both the open and office environment and on four different channels for the one-bedroom apartment. Multiple (ranging from 2 to 4) targets entered the area one by one and walked along predefined paths. This was done for both intersecting and non-intersecting paths. The highest average tracking errors measured in the tests occurred in the complex office environment and were equal to 0.45 m for two targets, 0.46 m for three targets and 0.55 m for four targets. These results clearly demonstrated the value of this approach.

#### 3.1.5. Sub-GHz RTI

All of the RTI systems we have discussed so far operate in the 2.4 GHz ISM band. Due to the fact that 2.4 GHz is close to the resonance frequency of water, the human body will absorb a significant amount of radiation [69]. This causes additional attenuation of the received signal, which tends to make this frequency band very suitable for device-free localization of human targets. The use of other frequency bands could potentially offer interesting benefits, however. Sub-GHz frequencies, in particular, have been hypothesized to improve the energy efficiency of RTI systems [70] and be usable for larger environments given their increased range [71]. Furthermore, these frequencies generally have better penetration capabilities for through-wall scenarios. On the other hand, the impact of (primarily human) targets on the RSS values of the communication links is also likely to be less significant, which would mean that certain trade-offs will have to be made.

Before these aspects can be investigated in more detail, however, it is necessary to establish whether sub-GHz frequencies can be used in RTI at all. The first adaptation of an RTI algorithm for the 868 MHz band was shown in [70]. The authors of this study deployed an RF sensor network in two highly similar open indoor environments and attempted to estimate the location of a single stationary human target. They made use of a basic shadowing-based RTI algorithm with updated parameters to account for the different frequency bands. The most significant parameter to change was λ from the formula described in Equation (2), which defines the ellipse excess path length in the weight model. Given the larger wavelengths of the signals transmitted at these frequencies, a significant increase of this parameter was to be expected.

The technique that was implemented managed to achieve a maximum average localization error of 78 cm. This result indicated that the use of sub-GHz frequencies in RTI is viable. Furthermore, the authors compared different regularisation methods and established that a Tikhonov approach is superior to TSVD, thereby echoing the conclusions obtained in [59] for 2.4 GHz.

In [64], Jiménez et al. focused on the feasibility of another sub-GHz frequency band: 433 MHz. Given the more limited impact of human targets on the RSS measurements of a 433 MHz signal due to its longer wavelength (approximately 0.69 m), this feasibility was not at all intuitive. In addition to this sub-GHz research, the authors of this study also investigated the potential benefits of combining RTI and RSS-based trilateration. In other words, they proposed a combination of a tagged and a tagless localization system. For their setup, the authors made use of an active RFID system. 40 active RFID tags were placed on the wooden walls of a single room within an experimental home environment. The area surrounded by these tags had a size of 4 m by 4 m, approximately half of the living room. Each tag was placed at a height of 1 m and was capable of periodically (1 Hz) broadcasting a unique id and a status message. Additionally, 4 readers were installed in the room as well, with each reader being connected to two antennas. In total, this meant that there were 2 × 4 × 40 = 320 communication links from which RSS measurements could be obtained for the tagless localization system. The tagged system made use of RFID tags as well. Each target carried six tags in their pockets, leading to 2 × 4 × 6 = 48 links that could be used by the trilateration algorithm.

Aside from implementing basic shadowing-based RTI for 433 MHz, the authors also created their own non-imaging DFL algorithm. This algorithm was based on the use of a particle filter and consisted of a simple dispersive movement model combined with a link-shadowing measurement model that described the probability of measuring RSS attenuation in each link as a two-dimensional Gaussian distribution. Mean location estimation errors of respectively 0.48 m and 0.50 m were obtained for regular shadowing-based RTI (denoted by the authors as DFL-RTI) and their self-developed method (DFL-PF). The authors also applied these same algorithms to an (at the time) publicly available 2.4 GHz dataset from the SPAN Lab at the University of Utah [72]. This data was collected in a 6.2 m by 6.2 m open environment containing 378 Zigbee communication links. Mean localization accuracies of 0.25 m for DFL-RTI and 0.31 m for DFL-PF were obtained, with the DFL-RTI result perfectly matching the original RTI publication by Wilson and Patwari in which the SPAN data was used first [41].

The tagged localization algorithm was also particle-filter based and made use of the same dispersive movement model as in DFL-PF. Particle weights were updated based on a ranging model that incorporated a path-loss model, the parameters of which were determined by least-squares fitting to measurements that were collected by the active RFID system when the environment did not contain any targets. The performance of this tagged system was slightly worse than the DFL approach, with a mean error of 0.60 m. Interestingly, when finally combining both the tagless and the tagged PF-based systems, it was shown that this combination did not actually increase the accuracy when compared to the tagless system by itself. Only when the amount of reader antennas was drastically reduced to only three—thereby greatly decreasing the number of communication links—did the combination method provide a clear improvement.

In conclusion, the study clearly showed the feasibility of using the 433 MHz band in RF-based DFL systems. The use of the 2.4 GHz SPAN Lab data was also highly interesting, especially given the fact that the use of public datasets within this field of study has been very rare. This is an aspect that will be discussed in more detail in section V.

In [73], an attenuation-based system called redundant RTI was proposed which used both 868 MHz and 2.4 GHz signals. 14 nodes were installed in a complex indoor laboratory environment that contained many static objects. Each ’node’ consisted of four independent RF transceivers, two for 433 MHz and two for 868 MHz. Communication between different transceivers in the same frequency band was considered by the authors to occur on different communication channels (although this should not be confused with different frequency channels). Given that there were two frequency bands and that two-way communication occurred, up to 8 RSS values could be obtained from communication between two nodes. During the offline calibration phase, the ’channel’ and link filtering process took place based on fade level and reference RSS values for each remaining link were determined by taking the median of all communication on the remaining channels. This was performed separately for 868 MHz and 2.4 GHz.

During the online phase, regular shadowing-based RTI was performed based on data collected from the remaining channels and links. Separate image vectors were created for both frequency bands, after which they were combined through simple addition. Next, several empirically determined thresholding steps were applied to the resulting image to detect and locate human targets. An RMSE of 0.26 m was obtained when trying to locate a single human target, although in 18% of cases (5/28) the system failed to determine that there was only a single target present. A few tests with two stationary targets were also performed to establish the feasibility of this concept for potential multitracking systems, but this aspect was not formally investigated any further.

Aside from this multifrequency concept, the separate localization accuracies of each frequency band were also investigated and compared. Interestingly, RTI based on 868 MHz led to more accurate results than 2.4 GHz. A potential explanation lies in the fact that the longer wavelengths of the sub-GHz signals will be less affected by the myriad of static objects in the environments. These results were very promising for sub-GHz RTI.

We investigated the combination of multiple sub-GHz frequencies for RTI ourselves in [71]. 20 RF transceivers capable of communicating with each other through the DASH7 alliance protocol [56] were installed in an empty classroom environment of 60 m^2^. The nodes alternatingly communicated on the 433 and 868 MHz bands. Shadowing-based RTI was performed in order to locate a single stationary target. Separate RTI images were created for each frequency band and were subsequently combined through the use of two different methods.

The first approach consisted of simply adding the two image vectors as described in the previous paragraphs. The second method was probabilistic and made use of ’training’ RTI images. We performed several measurements for both 433 MHz and 868 MHz when a target was present in known locations. The attenuation values of the pixels in the immediate vicinity of these locations were collected. A separate collection was created for all other pixels that were located further away from the true location. Gaussian curves were fitted to both sets of data, which were then used to create a Bayesian model that could transform the attenuation images that the RTI algorithm produces into probability images. These probability images for 433 MHz and 868 MHz could easily be combined through elementwise multiplication. A thresholding method similar to the one used in redundant RTI was then used to detect and estimate an actual position of a target. This probabilistic method led to an RMSE of 0.54 m with only a 3% failure rate incorrectly estimating that only a single individual was present in the environment. The feasibility of a full-fledged RTI system that only made use of sub-GHz frequencies was strengthened by this result.

In [74], the same sub-GHz multifrequency algorithm was applied to a setup of 35 RTI nodes that were installed in a highly complex two-room office environment of 125 m^2^. This method was compared to a partial single-channel adaptation of flRTI [62] for 433 and 868 MHz. When estimating the location of a single, stationary target, RMSE values of respectively 0.95 m and 2.53 m were obtained for the Bayesian method and the flRTI adaptation. Furthermore, the Bayesian method led to an incorrect estimation of the number of targets present in 44% of all cases, while for flRTI this number was 27%. For two stationary targets, the RMSE for both methods was higher than 3 m. While a certain level of localization was still possible, the obtained results suggested that the selected approaches were too naive for very complex environments.

Finally, a highly original example of an 868 MHz RTI implementation—although the focus here did not lie specifically on the sub-GHz aspect—was the research performed by Wagner et al. in [75,76]. Instead of creating a WSN that comprises a multitude of active transceivers, the authors used a setup based on a commercially available passive bistatic RFID system. This had particularly interesting implications for the weighting matrix. The standard elliptical model did no longer sufficient due to the fact that communication within the passive RFID-based system consisted of two phases: a forward link which powered a transponder and a backward link representing the communication from the transponder to a reader antenna. A single RSS measurement could, therefore, be influenced by targets being present near the LoS of both links. This led to a slightly more complicated model in which only the pixels outside of two different ellipses corresponded to non-zero elements in the weighting matrix. The authors managed to obtain a mean localization error of 0.30 m for a target present within a 2.7 m × 2.7 m square surrounded by 36 passive transponders and showed the validity of their approach.

All of the aforementioned studies showed that the use of sub-GHz frequencies in RTI is viable. Much more research needs to be performed regarding this domain, however. A more robust method of combining multiple frequencies that does not require training data needs to be found. Next, more advanced systems need to be developed that can achieve high levels of accuracy in very complex environments. Furthermore, the two major hypothesized advantages of sub-GHz when compared to 2.4 GHz (energy efficiency and suitability for larger environments) need to be formally investigated.

#### 3.1.6. Adaptive RTI

In [77], Kaltiokallio et al. introduced an advanced RTI technique called adaptive radio tomographic imaging (ARTI). The main novelty of this technique was the fact that it was capable of automatically updating the RTI model parameters while the system was online. This was in stark contrast to all earlier RTI techniques which we have described thus far, in which spatial model parameters were determined during the offline phase. In some papers such as [62,65], the impact of certain parameters on system accuracy for a specific setup in a specific environment was experimentally determined and based on this information parameter optimization could take place. This takes quite a lot of time, however, and would be highly cumbersome for real-life applications. Furthermore, ARTI also introduced a smoothing method to automatically fine-tune the model even further.

Multiple experiments were performed with 2.4 GHz 802.15.4 transceivers in three different environments: a 70 m^2^ open area, a 58 m^2^ single-bedroom apartment and an 86 m^2^ lounge room in which the nodes were installed on the outside of the walls. The obtained accuracies when tracking a single individual (multi-tracking was deemed to be out-of-scope, although the authors noted that ARTI principles could also be applied to multi-tracking RTI approaches) were compared to an FLRTI implementation. Results indicated that the median accuracy doubled in comparison to FLRTI and even tripled when the smoothing method was applied as well. Despite these highly promising results, the authors noted that the estimators they used were relatively simplistic and indicated that there were many possible future research directions.

#### 3.1.7. Energy Efficient RTI

One important aspect of Radio Tomographic Imaging that has received significant research attention since shortly after the first shadowing-based papers were published, is the development of lightweight RTI systems. Lightweight in this context refers to systems whose primary goal is target tracking in an energy and/or computationally efficient manner. In this section, we will describe proposed systems that have these concerns as their main focus.

An interesting concept that is often mentioned in the context of energy- and computationally efficient RTI, is Compressed Sensing (CS). In 2009, Kanso et al. [78] introduced this concept for the first time to RTI. Compressed sensing is a technique that enables the reconstruction of signals that have been undersampled well below the expected Nyquist–Shannon rate, provided that these signals exhibit a certain level of sparsity. Because RTI images tend to contain only a small amount of pixels with a high level of attenuation, CS principles can be applied in this context and potentially lead to a reduction of the number of measurements (i.e., RF transmissions) that need to be performed. In their paper, the authors specifically investigated two existing sparse estimation techniques: ‘least absolute shrinkage and selection operator’ (LASSO) and ‘orthogonal matching pursuit’ (OMP). Their applicability was verified through the use of a simulation-based approach, with OMP being computationally less intensive but providing less optimal results. In addition to the compressed sensing aspect, the authors also studied the potential benefits of more decentralized RTI approaches in which the actual processing no longer took place in a single processing unit attached to some type of ‘main controller node’, but was actually spread out amongst the different nodes. They concluded that a decentralized approach was more robust to link- and server related failures, but was less efficient from an energy point of view. As a result, they hypothesized that an optimal system would combine elements from both approaches.

It is important to note, however, that the research described in the paper by Kanso et al. was oriented towards the actual imaging aspect of RTI. The RF-based imaging techniques we have described in our survey paper thus far used the imaging aspect as an intermediary step for target tracking, but a significant amount of research exists whose focus lies on imaging an environment in its entirety, including static objects and obstruction [79,80,81]. While we consider this topic to be generally out of scope, the introduction of CS to RTI by Kanso et al. has been influential for both imaging and localization-oriented research. Therefore, it could not have been excluded from a comprehensive survey of this field of study.

Ever since the publication of the first CS-RTI paper, much research has been performed regarding this topic and it is currently an active research direction within RTI (and DFL research in general). In [82], Wang et al. investigated the OMP algorithm as well and proposed a new signal reconstruction algorithm called Bayesian greedy matching pursuit (BGMP). The new algorithm was not only computationally lightweight but also seemed to be more accurate than both the OMP and the regular maximum a posteriori approach commonly used in RTI. Sparse Bayesian learning (SBL) was used by Huang et al. in order to adaptively determine the most optimal RF links from each new set of RTI measurements [83]. They continued their work in [84,85] and developed an enhanced Bayesian compressive sensing (BCS) algorithm for use in RTI which incorporated a heterogeneous noise variance learning system in order to reduce accuracy loss caused by multipath interference. Finally, in [86], Xu et al. combined the concept of spatial diversity as a result of using multiple antennas per node with compressive sensing techniques and managed to significantly outperform shadowing-based RTI, channel diversity RTI and the servo-node based version of flRTI.

An entirely different energy-efficient RTI concept was proposed in 2014 by Khaledi et al. [87]. In their system, communication within the RF network only occurred on links in the vicinity of the moving target. They considered two different methodologies: an ellipse-based approach and a radius-based approach. In the ellipse-based approach, links were only considered if their lines-of-sight were within an ellipse surrounding the current velocity vector of the target. The radius-based approach only included links that crossed a circle that had the current target location as its center and whose radius was adaptively determined. Both methodologies led to 50%–80% energy being saved without any particularly negative accuracy-related impact when compared to a multi-channel shadowing-based RTI approach. The radius-based approach even led to a slightly increased accuracy.

An overview of the most important radio tomographic Imaging techniques which we discussed in this entire subsection is provided in Table 2, Table 3 and Table 4.

### 3.2. Non-RTI Model-Based Methods

As can be gleaned from the name, all radio tomographic imaging techniques involve an image reconstruction step. An image vector is obtained and used to determine location estimates of entities in the environment. When locating a single target, one of the easiest methods to do so is to take the maximum of this image and treat it as a location measurement. A succession of these location measurements can then be combined with a motion model in order to create a full-fledged tracking system. This motion model typically consists of a Bayesian recursive filter (e.g., a Kalman filter as in [57,67]).

The use of the imaging step can be considered disadvantageous, however, because the discretization of the environment by dividing it into pixels leads to additional quantization error. In order to avoid this problem, several model-based tracking techniques have been proposed which do not include any imaging. Many of the approaches we will describe in the following paragraphs can be classified as ‘Bayesian DFL’ [88]. Entities are tracked directly without any intermediary imaging phase based on a model that describes the RSS-values as the result of the locations of these entities. This is then combined with a Bayesian filter to estimate these locations and their evolution over time.

Li et al. introduced sequential Monte Carlo radio-frequency tomographic tracking [89]. The technique makes use of a measurement model which describes the attenuation of a communication link as a function of a distance parameter λ. This parameter is highly similar to the one used in Equation (2) for the weighting model of most RTI implementations. It is related to the width of an ellipse with foci at the two nodes comprising a communication link whose edge passes through the location of a target. This measurement model is combined with a particle filter—also known as a sequential Monte Carlo method—to track a moving individual. One interesting aspect of this technique is the fact that several model parameters are regularly updated while the system is online. This is done through the use of an expectation-maximization (EM) procedure. One of these parameters describes the locations of the RF nodes, which are therefore not automatically assumed to be known (save for a few anchor nodes). This is an important difference when compared to most RTI techniques and can be considered a major advantage for situations where rapid deployment of a DFL system is needed. The technique does have a need for offline calibration measurements when the environment is entirely static, however, which somewhat limits its applicability. The validity of the technique was first demonstrated in [89] through a simulation-based approach. Real-life experiments were set up by Chen et al. [90] by installing 24 RF nodes in two open outdoor 7 m by 7 m environments of which one contained a tree in the middle while the other did not. Both the SMC-technique and a classic RTI algorithm combined with a Kalman filter were implemented and results were compared for tracking a single human walking a predefined path. Root mean square errors of respectively 0.49 m and 0.32 m for the tree and treeless environments were obtained by SMC. This was an improvement upon the classic RTI technique, whose RMSE values were equal to 0.86 m and 0.64 m.

Wilson et al. introduced another interesting DFL method which manages to bypass the imaging step and makes use of a fade-level skew-Laplace strength model [91]. The core idea underpinning this technique is the differentiation between communication links based on their static fade level. We have discussed this concept previously in the context of RTI-based multichannel approaches, but it was originally introduced to DFL in this paper. Fade level observations were used to construct a likelihood model based on the skew-Laplace distribution. According to this model, the distribution of RSS measurements of a link greatly depends on the static fade level and the position of a target relative to that link. This means that calibration measurements are necessary in order to determine the fade level of each link and obtain values for the parameters that are required by the model. Similar to the previous technique proposed by Li et al. [89], the model was then combined with a particle filter. The performance of this technique was evaluated through two experimental deployments of 34 RF nodes in different environments: a bookstore and a through-the-wall home environment. The average error when estimating the position of a stationary target was 0.83 m in the home environment. When tracking a moving target walking along a predefined path, the average error was 0.9 m in the home environment and 0.58 m in the bookstore environment. Interestingly, experiments were also performed for two moving targets in both environments. Here, the average errors were respectively 0.84 m and 1.1 m for the bookstore and the through-the-wall deployment.

Another non-imaging radio tomographic tracking system based on foreground detection was proposed by Zheng et al. [92]. The idea of this technique is to model the RSS distribution on each communication link as a mixture of two Gaussians: a low variance distribution and a high variance distribution. These distributions are respectively considered to represent ‘background’ and ‘foreground’ variance, in a somewhat similar manner as the concept of intrinsic and extrinsic motion in subspace variance-based RTI. Unlike subVRT, however, empty room calibration measurements are not necessary. Instead, an online learning algorithm is applied to update the parameters of the Gaussian mixture model. Using live measurements to update the mixture model of each link allows the system to detect the affected links in the network. As was the case in the two previously described non-imaging techniques, this information is combined with a particle filter to actually track a moving target. An experiment was performed with 24 RF nodes in a rectangular through-the-wall environment. A moving target walking along a predefined rectangular path could be tracked with an impressive RMSE of only 0.13 m. These results clearly showed the potential of this technique.

In 2013, Zhang et al. introduced a highly novel passive localization system called real-time, accurate, and scalable system (RASS) [93]. The basic building block of this system was a set of three communication nodes which were installed in a triangular manner in the ceiling of an indoor environment. Based on a (very limited) number of RSS training measurements, support vector regression (SVR) was used to create a full-fledged tracking model. Although the specific methodology was arguably related to passive fingerprinting (which we will discuss in the next subsection), we categorized RASS as a model-based technique due to the adaptive learning model it used which eliminated the need for manual fingerprint measurements at every possible location within the environment. Both the use of 2.4 GHz and 868 MHz was investigated for communication between the nodes, with 2.4 GHz being chosen due to its more sensitive RSS dynamics. Tracking accuracies of approximately 1 m were obtained within a triangular setup of which each side had a length of 4 m. Additionally, RASS had a particularly strong focus on scalability and tracking latency. Multiple triangular setups could be combined to form hexagonal ‘cells’. In an actual large-scale implementation, a multitude of cells would be installed in the environment with the nodes of a single cell-communicating on a separate frequency channel. In other words, nodes only communicated within their own cell, without the possibility of collisions occurring with packets sent within other cells. This led to a tracking latency within a cell that was experimentally shown to be bounded by approximately 0.26 m.

In 2014 Saeed et al. introduced Ichnaea [94], a Wi-Fi-based device-free localization system that focused specifically on requiring a limited amount of training overhead. The general concept behind their approach was the construction of a silence profile based on a short training period of approximately two minutes. This profile was then combined with statistical anomaly detection methods and a particle filter to obtain location estimations of moving targets within the environment. Experimental setups in three different test environments in which measurements were performed two weeks apart showed a maximum median error of 2.5 m and indicated that the technique was robust in regards to environmental changes.

Bayesian DFL approaches that make use of diffraction-based models to describe RSS- measurements as a function of target positions were developed by Savazzi et al. in [95] and Wang et al. in [96]. Savazzi et al. managed to obtain a sub-20 cm RMSE for tracking a single target in both open indoor and outdoor 20 m^2^ environments which contained 14 IEEE 902.15.4 nodes. Preliminary results from a setup in a much more complex multi-room indoor environment indicated that room-level accuracy was feasible. The specific approach of Wang et al. was experimentally tested in a relatively small open university environment measuring 3.52 m by 3.52 m. A total of 8 IEEE 802.15.4 nodes were placed around the edges of the environment at a height of approximately 1 m, with each node being connected to a panel antenna with a limited beamwidth in order to lessen the influence of reflections outside of the environment. Tracking RMSEs of 0.12 m and 0.11 m were obtained for two moving targets being present in the environment. Comparisons were made to an elliptical model (0.20 m and 0.18 m) which was originally developed by Wang et al. in [97] and an exponential Rayleigh model (0.17 m and 0.15 m) which was originally developed by Guo et al. in [98]. The diffraction-based approach outperformed both for a maximum of two moving targets being present in the environment. Shortly thereafter, Luo et al. [99] developed a methodology that combined the diffraction-based model with an exponential-Rayleigh approach. This led to optimal mass transfer (OMAT [100]) errors of 0.293 m and lower for experiments performed in three different environments: open outdoor (24 nodes—36 m^2^), open indoor (20 nodes—24 m^2^) and complex indoor (16 nodes—16 m^2^).

Another Bayesian DFL technique based on a three-state received signal strength model was proposed by Kaltiokallio et al. [101]. The core idea underpinning this method was the characterization of each communication link as belonging to one of three possible states: being influenced primarily by electronic noise when no target is near, being influenced primarily by reflections caused by nearby targets and being influenced primarily by shadowing loss when a target is standing directly in the line of sight. This concept was then combined with a particle filter to obtain an actual localization system. Experiments with a single (moving) target were performed in a series of small-scale corridors of different widths: 2.0 m, 3.0 m, and 3.5 m. Accuracy-related evaluation metrics were then compared to an implementation of the sequential Monte Carlo-radio-frequency radio tomographic tracking system we discussed earlier [89] and to an implementation of a DFL system based on an exponential-Rayleigh model [102]. Results indicated consistently better tracking accuracies for the three-state model, thereby clearly indicating the value of this approach.

A comparatively recent Bayesian DFL method developed by Hillyard et al. [103] made use of a new mixture model which estimated the probabilities of a link being affected by the presence of a (human) entity based on the entity’s distance to the link line. A particular novelty of this model was the fact that it explicitly took into account the possibility that a link could still be affected if this distance was quite large or that a link could remain unaffected even if the entity was directly present on the link line itself. The model was incorporated in two newly developed Bayesian localization methods called maximum likelihood localization (MLL) and hidden Markov model localization (HMML). This was then combined with a parameter update framework that could update model parameters without requiring labeled training data (i.e., empty room calibration or passive radio maps). Initial parameter values were estimated based on data collected by a human target walking around during the system setup phase, after which a process of continuous recalibration took place while the system was active. The resulting system was capable of locating both a stationary and a moving target, did not require an intensive labeled training period and could adapt to environmental changes. This final aspect, in particular, is currently a major issue for DFL-techniques which makes use of a passive radio map, which is a topic we will discuss in more detail in the next subsection.

The proposed system was experimentally validated in three different environments (empty classroom, furnished floor of a home and furnished basement) in which RSS data was collected for over seven days. Comparisons were made to regular RTI, KRTI and a passive fingerprinting approach which made use of linear discriminant analysis (LDA). Results indicated both MLL and HMML were capable of locating a single target without any accuracy losses caused by both intentional and unintentional environmental changes. Furthermore, localization errors were reduced by 11 to 51% when compared to the other DFL approaches.

The concept of compressed sensing (CS) which we discussed in the previous RTI-focused subsection, has in recent years been gaining popularity for non-RTI algorithms as well. In 2015, Wang et al. [104] proposed an interesting CS-based DFL algorithm which did not only make use of multiple frequency channels but of multiple transmission power levels as well. Using a 2.4 GHz RF network which consisted of only eight measurement nodes (and one controller node), their approach managed to obtain sub-meter accuracies in both a 64 m^2^ open outdoor and a 36 m^2^ complex indoor office environment. Furthermore, it was capable of multitracking, as evidenced by experiments in which two targets were simultaneously present in an outdoor open area.

Another CS-based DFL method called E-HIPA (’Energy-efficient framework for high-precision multi-target-adaptive device-free localization’) was proposed by Wang et al. [105] in 2017. As can be gleaned from the name, the entire focus of this methodology was energy efficiency and they managed to obtain an energy decrease of up to 69% with meter-level multi-tracking localization accuracy when compared to implementations of the RTI, SCPL, and RASS-algorithms. Energy-efficiency was the main focus of the Bayesian DFL approach described in [88] as well. In this paper, the authors did not implement CS-based techniques, however. Instead, they drastically reduced the amount of transmissions performed by the transceiver nodes by having nodes only communicate a binary link-state (link obstructed or unobstructed) to a central base station instead of RAW RSS-measurements. Transmissions only occurred when a link was obstructed.

An interesting DFL tracking method that uses an imaging-based approach that is clearly distinct from RTI was proposed by Wang et al. [106]. The main novelty of this research was the use of a Bayesian grid approach (BGA) in which the observation information of the shadowed links, the constraint information of the non-shadowed links and the prior information from the previous estimation was combined. A prominent feature of this BGA was its computational lightweightedness, requiring only a small amount of grid multiplication and addition operations. The authors explicitly contrasted this with the matrix inversion in RTI, although it should be noted that—as stated in previous sections—this inversion should only occur during the offline creation of the projection matrix.

Experiments with this technique were performed in an open environment of size 8 m by 8 m, at the edges of which 17 IEEE 802.15.4 nodes were installed. One node acted as the central node while the other 16 collected the necessary RSS measurements. The resulting mean accuracy when tracking a single moving target was equal to 0.155 m. Comparisons to implementations of the fade-level skew-Laplace model [91] (0.443 m) and a compressive-sensing based RTI technique [107] (0.322 m) were made, clearly outperforming them both. Additionally, the average running times of the three algorithms were compared on a regular PC containing a 2.4 GHz processor. These running times were equal to 1.5 ms for BGA, 6.9 ms for the Fade-level based technique and 116 ms for the CS-based RTI. Based on these promising results, it was concluded that the proposed approach was suitable for use in contexts in which computational resources were limited.

All of the research techniques described in this subsection so far made solely made use of RSS measurements. CSI-based techniques for tracking applications have been developed as well, however. In 2016, Wang et al. proposed one such DFL method called LiFS [108]. LiFS made use of a pre-processing step in which raw CSI measurements were filtered to only include subcarriers that were influenced the least by multipath effects. The remaining measurement data was then used as input to a model that consisted of a set of power fading-based equations in order to determine target locations. Not only did this approach not require any prior training, it was also capable of multitracking and obtained a better localization accuracy than Pilot [109] (a fingerprinting technique which we will discuss in the next subsection), RASS and RTI in three complex indoor environments. Furthermore, the technique did not require that the locations of all Wi-Fi transceivers in the environment were known. An overview of the most important non-RTI model-based tracking techniques which we discussed in this subsection is provided in Table 5.

### 3.3. Passive Radio Mapping

#### 3.3.1. Early Fingerprinting System

As mentioned in the previous section, when Youssef et al. formally defined the research domain of device-free (passive) localization in [6], they also showed the feasibility of the concept itself by constructing an indoor DFL system. This system was capable of detecting and tracking a single human intruder. It consisted of two 802.11b access points (AP) which periodically broadcasted packets and two wireless sniffers which acted as monitoring points (MP). In total, this meant that there were only four one-way communication links that could be used to detect and track potential targets.

Tracking in this DFL system was performed through the use of a passive radio map. Radio maps are a well-known concept in fingerprint-based active localization systems [110,111], where they are constructed by having a tagged target stand at certain predefined radio map locations during an offline phase. Signal strength characteristics of the communication links between the tag and a series of static nodes in the environment are collected and stored in a fingerprint database. This database is the actual ‘radio map’. When the system is active, real-time measurements are compared to the database and the best matches are assumed to correspond to the fingerprint locations closest to the real location of the target.

**Figure 7:**
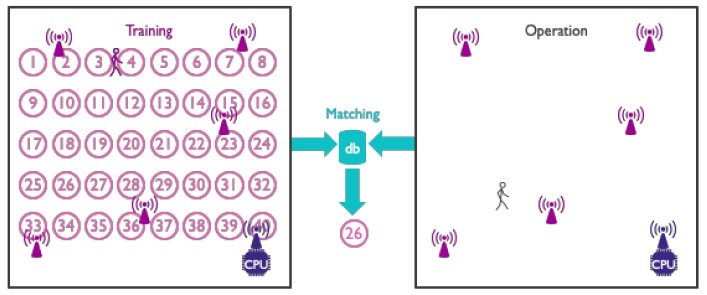
Schematic overview of passive radio mapping.

**Figure 8:**
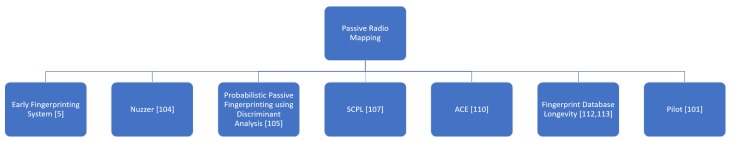
A schematic overview of the main passive radio mapping approaches discussed in this survey.

The concept behind passive radio maps (or ‘passive fingerprinting’) is similar. Rather than relying on the communication links between a tag and static nodes in the environment, however, the communication between the static nodes themselves is used. For each fingerprint location where a human individual is present, a (hopefully) unique RSS pattern will emerge between the static nodes. This pattern is saved to a database that is used during live measurements to estimate a location. In the vast majority of cases, actual measurements are performed to create the passive radio maps, although it should be noted that some very limited research exists in which propagation-model based simulations are used [7,112]. The concept of passive fingerprinting is schematically illustrated in Figure 7 and an overview of the techniques we will discuss in this subsection is provided in Figure 8.

In the system described by Youssef et al., four fingerprint locations were determined. In the radio map, each location was associated with a specific set of RSS histograms of all four communication links. ‘Live’ measurements when a target was present in one of the four possible locations were compared to the radio map histograms through the use of a simple Bayesian-inversion based inference algorithm. The most likely match was then considered by the system to correspond to the true location.

Two identical experiments were performed in which a target walked into the environment and stood still at four different predefined positions for approximately 60 s while measurements were being performed. These positions were spaced 0.91 m apart. The data from both experiments was alternatingly used for constructing the radio map and for evaluation. A correct estimate of the true location was obtained in respectively 86.3% and 89.7% of cases, depending on which dataset was used for training. The corresponding average localization errors were equal to 0.21 m and 0.16 m.

While this DFL system was clearly very limited in scope, the fact that a simple implementation managed to obtain such accurate location estimates readily demonstrated the potential of this technique.

#### 3.3.2. Nuzzer

In [113], Seifeldin and Youssef demonstrated a large-scale passive fingerprinting system called Nuzzer. It made use of the same concepts as the basic system described in the previous paragraphs and investigated both single- and multitarget environment. For the single target case, the authors’ primary test environment consisted of an entire floor in an office building with an area of 1500 m^2^. Two laptops served as MP’s and captured packets sent by three 802.11 b AP’s that were already present in the environment and did not need to be installed. As a result, only six one-way communication links could be used to estimate the location of a target. A probabilistic Bayesian-based inference algorithm was used to determine the passive radiomap locations that were most likely to correspond to the actual location of the target. The performance of this technique was compared to two other location estimators: a random estimator and a deterministic method. The deterministic method made use of a very simple radiomap which did not contain RSS histograms but instead utilized average signal strength vectors. A location was then estimated by determining which of these vectors lay closest in signal strength space to an online measurement vector.

After the Bayesian fingerprint matching has occurred, spatial and temporal averaging techniques were applied to the *n* most likely discrete locations in order to obtain a location estimate in continuous space. Results indicated a median accuracy of 1.82 m, while the continuous space estimator for the deterministic method obtained a median accuracy of 6.74 m. A much smaller (130 m^2^) floor of an office building was used as a second test environment for the single target case. Nuzzer obtained a median localization error of 0.85 m, whose significant decrease compared to the previous experiment was—given the smaller size of the test environment—in line with the expectations of the authors.

For the analysis of a multi-target case, two different test environments were used as well. The first environment was an indoor 24 m^2^ curved corridor which contained 3 APs and 4 MPs. It was subdivided into 5 different zones based on the locations of the LoS of the 12 communication links. The general idea behind the multi-target approach was to first detect the number of targets present by making use of the average relative variance measured over all communication links. Thresholding on this value could provide a reasonably accurate estimate, with the method being correct in 81% of all cases within this environment. Next, the same relative variance-based approach was utilized on individual links to determine for each zone separately if it contained a target. This approach was accurate in 80% of the measured cases. Finally, if a single target was located within a zone, a regular single-target fingerprinting approach could then be used to determine their location. No quantitative information was provided regarding the accuracy of this final step, but the proposed approach indicated that some level of multi-tracking with passive fingerprinting was at least feasible. The second experiment took place in a 130 m^2^ office environment and was subdivided into 6 zones based on logical areas (e.g., hallway, separate rooms, ...). As expected, the accuracy of the target estimation (71%) and zone detection (61%) was much lower in the larger environment, but these results still showed that the concept was feasible.

In conclusion, it could be stated that the passive Fingerprinting-based Nuzzer system managed to obtain highly impressive passive localization results in very large environments with a low amount of communication links. Additionally, some level of multi-tracking was shown to be feasible as well, although it must be stated that this aspect still required a significant amount of further research (e.g., what if multiple people are present within the same zone?). Nevertheless, as one of the first robust passive fingerprinting systems, Nuzzer represented a major step forward within this field of research.

#### 3.3.3. Probabilistic Passive Fingerprinting Using Discriminant Analysis

Xu et al. developed a passive fingerprinting technique that utilized probabilistic classification approaches based on discriminant analysis [114]. The authors compared three different discriminant analysis methods for matching a live measurement to the most likely passive radio map location: minimum euclidean distance (MED), linear discriminant analysis (LDA) and quadratic discriminant analysis (QDA). The ‘MED’-approach is highly similar to the deterministic estimator we discussed earlier in the context of the Nuzzer system. In LDA, the goal is to find a linear combination of features which can be used to differentiate between multiple classes. The density of each class was assumed to be a multivariate Gaussian with a unique mean and a common covariance matrix. A discriminant function was established and maximized to find the most likely class to which a live measurement vector belongs. QDA is a generalization of LDA which allows for different covariance matrices. While this leads to an increase in flexibility, it also leads to a huge increase regarding the number of parameters that need to be estimated. For both LDA and QDA, the RSS difference vectors could be projected to their first q principal discriminant components to reduce the computational overhead caused by parameter estimation. As indicated by the authors, a more exhaustive explanation of discriminant analysis can be found in [115].

The authors installed RF devices capable of communicating in the unlicensed 433.1 MHz and 909.1 MHz bands in two different environments: a one-bedroom apartment (∼40 m^2^) and a much larger, complex office space (∼150 m^2^). Results showed the LDA-approach consistently outperforming the two other methodologies for tracking a single human target. Additionally, the use of the 433.1 MHz frequency band led to increased accuracy as well. The authors hypothesized that this was due to its larger wavelength causing smoother RSS-variations when influenced by moving human targets. Another important aspect that the authors investigated, was the amount of time during which the reference database still remained valid. Changes in the environment or hardware-related issues such as radio drift can potentially lead to a severe decline in system accuracy if the fingerprints are not updated accordingly. Two different methodologies based on the use of a bias vector were proposed to extend the database lifetime. Results indicated a cell estimation accuracy of 80% with a month-old fingerprint database using the naive correction approach and 90% with the truncated correction approach. If no correction was applied, an accuracy of approximately 20% was observed. A continuation of this research by Xu et al. [116] proposed an even more improved automatic re-calibration approach that incorporated data from auxiliary sensors indicating the status of doors (open/closed) and chairs (occupied/unoccupied).

Finally, the potential of using this system to locate multiple entities was investigated. The number of targets n was assumed to be known and then most likely classes to which an active measurement belonged were considered to correspond to the correct cell locations. This relatively simple method still resulted in an impressive cell estimation accuracy of 83.5% and an average localization error of 0.89 m when estimating the location of three stationary targets.

In conclusion, the proposed passive fingerprinting method based on discriminant analysis showed promising results. The use of LDA led to a significant increase in accuracy when compared to a more classic, deterministic approach. Furthermore, this work was one of the first passive fingerprinting studies which investigated the possibility of locating multiple targets.

#### 3.3.4. SCPL

Xu et al. continued their research into passive fingerprinting and proposed a new algorithm called sequential counting, parallel localizing (SCPL) in [117]. As can be gleaned from the name, this system consists of two separate phases while it is active: counting the number of targets present and estimating their locations. Despite the fact that SCPL is focused on multi-tracking, this fingerprint database still only contains measurements that were obtained while a single target was present in the environment. Using measurements for all possible combinations of locations for n targets would increase training overhead by such an extent that it would be essentially impossible to collect the required data.

SCPL uses the total energy change in the environment Υ as a crucial metric in the counting phase of the algorithm. This metric is defined as the sum of the RSS changes of each individual link. The assumption is made that the presence of multiple subjects in an environment will both affect a larger number of spatially distributed communication links and will also cause larger RSS changes on these links. Υ-values calculated from the RSS data that was collected for the construction of the fingerprint database can be used to determine whether the environment is empty, contains a single individual or contains multiple targets. Υ in and by itself is not sufficient for providing an exact count, however, as it is not linearly proportional to the number of subjects. Instead, a successive cancellation approach which consists of multiple rounds is used in order to count the targets. During each round, a regular probabilistic method is used to determine the most likely cell location if only a single target were present. The corresponding mean RSS difference vector for that cell in the fingerprint database is then subtracted from the measured RSS difference vector and the resulting vector is used in the next round. This process continues until the energy change is below a certain threshold. Based on the number of subtractions, a count is obtained. As stated before, however, the problem is not linear. Therefore, the RSS difference vector from the fingerprint database is first multiplied by a coefficient before it is subtracted from the measured RSS difference vector. This coefficient is unique for each link-cell combination. A formal description of the algorithms for both the calculation of this coefficient as well as for the full counting phase are provided by the authors in [117].

Once the number of targets has been determined, the tracking phase occurs in which their locations are estimated over time. The trajectory of each target is modeled as a state transition process under a conditional random field (CRF) [118]. A sensor model and a transition model are defined, after which the most likely consecutive set of state transitions is determined based on the Viterbi algorithm [119]. Environmental constraints (e.g., walls) for human movement were taken into account as well.

The authors performed experiments in two different environments: an office setting with a total area of 150 m^2^ and an open floor space of 400 m^2^. Both counting percentage and error distance were used as evaluation metrics and four different scenarios for subject counts of 1 to 4 were investigated for each environment. Results indicated an average localization error of approximately 1.08 m in the office environment, with significant differences depending on whether the subjects walking trajectories overlapped. For the open floor environment, a significant percentage of nodes were installed on a wall that contained a large amount of metallic parts, thereby severely inhibiting radio propagation. Despite this particular difficulty and the much larger environment, fairly accurately localization results were still obtained: an average counting percentage of 86% and an average localization accuracy of 1.49 m. These results indicated that a passive fingerprinting based approach was clearly feasible for multi-tracking without requiring an unfeasible amount of training overhead.

#### 3.3.5. ACE

The aforementioned feasibility became even more clear with the development of the multi-tracking ACE localization system by Sabek et al. [120]. At its core, ACE made use of an energy-minimization framework. The energy function in this framework was represented as the sum of three terms: a temporal prior term, a spatial prior term, and a likelihood term. This likelihood term indicated the likelihood of each possible location corresponding to an actual target location based on the measured RSS patterns. The actual minimization of the energy function then occurred by mapping the problem to a binary graph-cut problem. The output of this operation consisted of potential target locations. Next, a hierarchical clustering algorithm was applied to the candidate locations and the centers of mass of the resulting clusters were considered to correspond to the actual target locations.

In order for the energy minimization framework to perform its task, however, it needed an estimate of both the RSS likelihood and the temporal transition priors. Both could be obtained during the training phase when the system was offline. Fingerprints in ACE were collected using a cross-calibration technique in which only one human target was required to be present in the environment during the training phase. The general idea of this technique was that each potential location was associated with two different histograms: an ‘active’ histogram containing measurements when the location was occupied by a target and a ‘passive’ histogram containing measurements when the location was empty. The passive histogram for a certain location contained the measured RSS values from the active histograms of all other locations. This methodology enabled the possibility of multi-tracking without requiring a manual collection of fingerprint data for each possible combination of target locations.

Experiments to evaluate the capabilities of ACE were performed in two different test environments: a 114 m^2^ residential apartment and a 130 m^2^ office environment. In both environments, 2 AP’s and 3 MP’s were installed, leading to a total of six (one-way) RSS links that could be measured. Additionally, up to three human targets could be present in the environment during the online measurements. The performance of ACE was compared to the Nuzzer and SCPL systems discussed earlier, as well as to Spot [121], an early version of ACE with a less pronounced focus on multi-tracking. Median errors of respectively 1.33 m and 1.43 m were obtained in the residential apartment and office environment when a single target was present. When including location estimations from all possible number of targets (1–3), the errors increased to 2.11 m and 1.44 m. This represented an enhanced accuracy of at least 11.81% when compared to the other techniques. The second ’best’ technique in terms of accuracy appeared to be SCPL, with median error values of 2.42 m and 1.61 m. However, the average running times per location estimate of ACE were in the range of 2–3 ms, while for SCPL these could take up to 610 ms. These results clearly indicated the viability of the ACE system for multitracking DFL applications.

#### 3.3.6. Fingerprint Database Longevity

Despite the impressive passive fingerprinting results that were shown in the previous paragraphs, there is still one important downside upon which we have touched only lightly thus far: database longevity. This aspect was briefly discussed by Xu et al. in [114] when they developed two methodologies based on the use of bias vectors to increase the amount of time for a fingerprint database to still be viable. However, the true extent of the problem in regards to how sensitive passive fingerprinting truly is to environmental changes only became clear after the experimental work performed by Mager et al. in [122].

In this study, the authors installed a network of 30 transceivers in a 84 m^2^ complex home environment comprising a living room, dining room, kitchen, bedroom, den, and two bathrooms. These transceivers were placed pairwise in 15 locations at heights of respectively 28 cm and 132 cm above the floor. They communicated on 8 different channels spaced at least 10 MHz apart within the 2.4 GHz range. 32 separate locations that were used for both training and evaluation of a passive fingerprint system with a single target being present were defined and marked by numbers on the floor. The main novelty of their study was the approach the authors utilized to simulate the manner in which environments change over time. They identified a set of movable objects (a couch, a bag of groceries, a dining set, etc.) and assigned each object a list of four possible locations or states. From this list, a default state was selected which indicated the position and/or orientation of the object during the training phase. During the evaluation phase, the state of a single object would be randomly changed at the beginning of each successive experiment. Changes were cumulative and therefore the environment would differ more and more from its initial state as the number of the experiment increased.

For constructing the actual fingerprinting system which compared live measurements to a fingerprint database, the authors investigated four different classification techniques: k-nearest neighbors (KNN), support vector machine, LDA and random forests. Random forests clearly outperformed all other approaches, with LDA and KNN leading to particularly inaccurate location estimates for this setup. Furthermore, when investigating the impact of static location changes, results indicated the error rate doubled on average over every 6.1 minor furniture changes. Only 3.9 changes were necessary to double the RMSE. The use of a multi-channel approach with eight different frequencies channels which made use of a fade-level based selection algorithm severely improved the database longevity. This work was expanded upon by Lei et al. in [123], who performed additional analysis on the same data sets and managed to improve slightly upon this technique by introducing an enhanced channel selection algorithm. Furthermore, they demonstrated that the use of a logistic regression classifier outperformed random forests. Nevertheless, the fundamental problem of fingerprint database longevity was not truly solved and it still remains an important challenge to overcome.

#### 3.3.7. Pilot

All of the passive fingerprinting systems we have discussed so far make solely the use of RSS measurements. Given the benefits that the use of CSI has brought to many DFL systems as outlined previously, it is not surprising that CSI-based fingerprinting systems exist as well. In 2013, Xiao et al. introduced Pilot [109], a passive radio mapping approach that made use of the CSI sensing modality. Their approach consisted of three separate procedures: passive radio map construction, anomaly detection, and position estimation.

The two goals of the passive radio map construction step were the creation of a ‘normal’ CSI profile that could be used for anomaly detection and the creation of an actual fingerprint database mapping measurements to potential target locations. The specific CSI derived feature that was used in the entire Pilot system was the averaged self-correlation of the measured CSI value of a single packet with all other packets. For the normal profile construction, self-correlation values of a set of obtained CSI measurements were collected when the environment did not contain any targets. Similarly, the actual fingerprint database was created by collecting self-correlation values of CSIs for a target being present at each potential location. Each communication link within the system was considered to be indicative of a specific subsection of the environment (its ‘sensing zone’) and had its own separate normal profile and fingerprint database. The shapes and sizes of these sensing zones were individually determined for each link based on cross-correlation calculations between the normal profile and fingerprint database CSIs. If locations existed that did not fall within any sensing zone (‘dead spots’), targets present in these locations could not be detected or tracked. This issue could be resolved by manually optimizing the placement of the transmitters and/or receivers.

In the anomaly detection phase, live CSI correlation measurements were compared to the normal profile created in the previous step. An anomaly (i.e., the presence of a target) was detected based on a kernel density estimation combined with a thresholding approach. Upon this detection, the location of the target was determined in the position estimation step. A kernel density-based maximum a posteriori algorithm was used to determine the most likely target location in the database which matched the measurements. It should be noted that it was possible for a target to be present in overlapping sensing zones. In this case, the entire process described thus far was performed separately for each link and the final location estimate was obtained by determining the position that maximized the joint possibility. The potential locations considered for fingerprint matching were also limited to those within the overlapping area. The system only took into account the tracking of a single target and anomaly detection on multiple links was automatically assumed to indicate target presence in the overlapping zone.

Experiments were performed in two indoor testbeds: a 7 m by 11 m laboratory environment and a large lobby environment of approximately 776 m^2^. Two 802.11n APs (WR941ND router) and MPs (NIC in a standard HP laptop) were installed in each testbed, leading to a maximum of four communication links. Each device only made use of a single antenna, with the authors noting that a multi-antenna setup would be left for future work. First of all, the accuracy of the anomaly detection was assessed by comparing its performance to an implementation of the RASID system [44] which we have discussed previously in Section 2. A thresholding aspect was present in both Pilot and RASID and in both cases, the selection of the value(s) of the relevant parameter(s) came down to a trade-off between the detection rate and the false positive rate. For a false detection rate of 30% or lower, the detection rate of Pilot greatly outperformed RASID in the Lab environment (approximately 60% for RASID while well above 80% for Pilot). Similarly, the lobby environment indicated superior performance by Pilot as well. These results seemed to indicate a clear detection advantage for the CSI-based Pilot in comparison to the RSS-based RASID.

For the actual position estimation, a comparison was made to a Nuzzer(-like) system, which we have discussed earlier as well. Pilot outperformed Nuzzer in every single test scenario, with the location estimation accuracy of Pilot at one point reaching up to 98%. While the differences were relatively small when the systems used only a single link, they became particularly pronounced when all four available links were utilized. Similarly, increasing the number of samples used for the creation of the fingerprint database had a much more significant impact (an accuracy increase of more than 10%) on Pilot than on the Nuzzer-like system.

In conclusion, this research clearly showed the potential of CSI-based passive fingerprinting systems. While there were (and currently are) still quite a lot of research problems to be solved (multi-tracking, database longevity, potential benefits of multiple antennas, etc.), this was nevertheless an important step towards the use of CSI not only for detection but for tracking as well.

An overview of the most important passive fingerprinting techniques which we discussed in this subsection is provided in Table 6.

## 4. Identification

As we have mentioned before, solving the ‘identification problem’ with an RF-based solution is a major challenge in the field of device-free localization. Differentiating between multiple subjects based on the subject’s influence on RF links remains a difficult task and only in recent years have there been several attempts to do so. In [124], a low-cost traffic analysis system called ‘monitor’ was proposed which made use of RSS measurements from a very limited number of RF links. It was capable of differentiating between human subjects and cars. Given the differences in materials, size, and geometry, the impact on RF waves was noticeably different. Despite its relative simplicity, this system was an important first step towards a full-fledged solution. Another highly interesting vehicle-based study was performed by Anderson et al. in [125]. They investigated an RTI-based method to detect and potentially differentiate between different types of vehicles. Experiments performed with a small electric car, a large passenger car, a large cargo van, and a large school bus strongly indicated the possibility of doing so, provided that the vehicles were clearly differently sized. In the next few subsections, we will discuss several techniques that have been developed to differentiate between human subjects.

### 4.1. RSS-Based Fingerprinting

In 2015, Scholz et al. proposed WiDisc [7], which was one of the first RF systems capable of differentiating between multiple human targets. It could distinguish between three human subject classes (tall, medium and small) and solely made use of RSS measurements. The system took a passive radio mapping approach in which a fingerprint database contained fingerprints for each possible combination of a location and a type of subject. Interestingly, fingerprints could be collected both manually or through simulation. The authors’ simulation-based approach made use of 3D subject models which were created by using the skeleton tracker capabilities of a Microsoft Kinect system. The skeleton tracker output for each test subject was used as input to create a relatively simple model consisting of several 3D-boxes. These models were then used in a simulated representation of the real environment where a wave propagation model was used to approximate actual fingerprint measurements. The goal of this simulation-based approach was to investigate its potential for eliminating manual training overhead.

The fingerprinting methodology of WiDisc was deterministic and based on distances between measurement vectors and fingerprints. It should be noted that—despite the fact that the fingerprint database contained either simulated or measured data for multiple locations—the output of this module solely estimated the subject class of the target present. Identification was considered to be the sole focus of this research. Experiments with WiDisc were performed by the authors in a 4.04 m by 5.33 m by 2.70 m lab environment which contained a small amount of furniture. Four IEEE 802.15.4 transceivers that communicated on three different carrier frequencies within the 2.4 GHz band were installed in the lab at a height of approximately 0.80 m. The RSS-measurements of these three channels were averaged for each link. 3D-model simulations and actual measurements were performed with three different subjects (a child, a woman, and a tall man) being present in the environment at seven predefined locations. During an actual measurement, a subject stood still at a location for a duration of 5 s. The resulting average subject estimation accuracy when using a fingerprint database based entirely on simulation was 67%. There were clear differences in the accuracy depending on the actual subject class: ‘small’ was estimated correctly 43% of the time, ‘medium’ 71% of the time and ‘tall’ 86% of the time. The authors suspected that the particularly poor estimation of the ‘small’ class was related to the limited height of the subject (0.94 m, barely reaching the LoS of the nodes) and the inherent motion of a three-year-old child. The use of a manually acquired fingerprint database based on actual measurements led to a relatively small accuracy increase to 76%. While the authors acknowledged that WiDisc still needed further development, it nevertheless managed to obtain impressive results and was one of the first purely RF-based attempts of solving the identification problem.

### 4.2. CSI-Based Gait Analysis

As was the case for multiple detection- and tracking-oriented systems, the use of CSI has also proven to be very beneficial for identification. An approach that has become very popular over the past two years consists of using Channel State Information from a Wi-Fi network to characterize the gait of a human target in the environment. Gait has been known for over a decade to be a unique feature of a person and therefore a prime candidate for differentiation between multiple targets [126,127]. Zhang et al. proposed WiFi-ID [128] which extracted unique features from the CSI that are representative of the walking style. The system was capable of correctly identifying a person from a possible list of 2 to 6 people with an accuracy of respectively 93% to 77%. The WiWho framework introduced by Zeng et al. in [129] achieved similar results with accuracies of respectively 92% to 80%. FreeSense [130] was an approach by Xin et al. which made use of a combination of principal component analysis, discrete wavelet transform and dynamic time warping. A FreeSense system installed in a 30 m^2^ smart home environment obtained accuracies of 94.5% to 88.9% for candidate user sets of sizes 2–6.

One of the most recent systems utilizing this principle called Wii [8] was introduced by Lv et al. in 2017. Wii could calculate the periodicity from the CSI of Wi-Fi signals influenced by human movements and could extract time—and frequency—domain features from both single and multiple steps by the target. This approach led to superior identification accuracies. Additionally, the system was capable of recognizing ‘strangers’ which were not part of the candidate user set.

The framework of Wii consisted of four main components: pre-processing, step segmentation, feature extraction, and classification. CSI data was collected from a single IEEE 802.11n link between a commercially available wireless router and a laptop containing a three-antenna Intel Wi-Fi Link 5300 network interface card which provides access to CSI data. The first step of the pre-processing stage consisted of a linear interpolation in the collected CSIs to calibrate the sampling frequency in case the number of collected CSIs did not match the number of packets sent by the router. For each packet, a CSI matrix of size 3 × 30 (30 OFDM subcarriers) could be obtained. Despite the fact that the subcarriers in OFDM are selected so that they are all independent, some relationships did exist in CSI space between adjacent carriers. In order to obtain independent data, principal component analysis was used. The 3 × 30 matrices were reshaped to 1 × 90 CSI vectors which were then concatenated for all vectors obtained during a fixed time interval. The result was an n × 90 matrix of which the principal components were calculated. Due to the generally high contribution rate of the first principal component (>85%), it was used as the representation of the collected data. Next, a low-pass filter with a cutoff frequency of 10 Hz was applied to the resulting signal to filter out noise related to non-walking movements. The output of the filter was then sent to the step segmentation stage.

The goal of this stage was to find the start and endpoint of each human step movement from the collected data. A combination of a continuous wavelet transform (CWT) and wavelet variance was used to discover periodicity within the signal. Once the signal could be easily subdivided into different human step movements, it was sent to the next stage. This stage was responsible for feature extraction. Both time domain and frequency domain features had to be calculated within a certain window of the signal. Two different types of windows were used: step windows that contained the signal for the duration of a single step and walking windows which contained multiple steps. An analysis of the normalized information gain of 11 predefined features was performed by the authors to determine the five features that were actually used in the live system. For the step windows, maximum, minimum and standard deviation were used as time-domain features and entropy was used as a frequency domain feature. For the walking windows, only average step time was used as a time-domain feature.

The resulting features were stored in a vector and sent to the part of the framework which was responsible for the actual classification (and the required training during the offline phase). This stage consisted of two parts: a Gaussian mixture model (GMM) to differentiate between known human profiles and strangers and a multi-class support vector machine (SVM) with a radial base function (RBF) kernel to differentiate between known humans. Finally, it was also possible in Wii to perform some post-processing on the raw classification results of the final step (e.g., to take into account the fact that successive steps in a walking window will very likely belong to the same person).

The proposed system was evaluated in a meeting room environment of size 5 m by 4 m. A single AP–MP pair was installed in the environment at a height of approximately 0.75 m. Human targets would enter the room (without any other target being present) and walk naturally along a predefined path that crossed the LoS of the link. Eight human volunteers were used for the entirety of the experiments. For each evaluation, a holdout cross-validation approach of five steps was performed and the results were averaged.

Extensive evaluations were performed to optimize certain parameters such as the size of the training sets (in steps) and the number of steps included in a walking window. The stranger recognition capability was evaluated as well and could obtain accuracies between 90% and 95%, provided that the number of strangers in the training set was not too large. Furthermore, direct comparisons to the previously mentioned WiWho and Freesense systems were also performed. Wii obtained average identification accuracies ranging from 98.7% to 90.9% for candidate group sizes of 2 to 8 humans. These were markedly better results than WiWho (90–95% to 65–70%) and FreeSense (90–95% to 75–80%).

Interestingly, the authors also noticed some clear differences in identification accuracy depending on the actual target, varying from 83% to 95% in the full group case in which all eight volunteers were potential candidates. Volunteers with a similar height and weight tended to be more difficult to differentiate, while the only two female volunteers and a very tall male volunteer had identification accuracies of 94–95%.

While these results were most certainly impressive, the authors noted several limitations of the system which could inspire future research directions. First of all, both the training and the evaluation data had to be collected when the target was walking along the same, predetermined path which clearly intersected the LoS of the RF link. Second, the targets had to be constantly walking using their natural gait. Finally, Wii could be only used when there was only one human target present in the environment. Nevertheless, Wii was shown to be an effective tagless identification system and its development contributed significantly to an eventual solution to the identification problem.

One potential solution to the problem of live subjects not walking along a predefined path was to increase the modality of the system. In [9], Chen et al. proposed ‘Rapid’, which combined a CSI-based approach with acoustic information from footstep sound. The main idea of this system was to create a channel frequency response power variance-distance model that quantified the distance between the actual walking path and the LoS path of the communication link. This value could then be used to indicate system noise. If a high amount of system noise was discovered, the additional acoustic sensing modality was brought in to complement the identity estimation. The value of this approach was demonstrated in three different environments: a 2 m wide corridor in a university building, a 9 m by 6 m laboratory environment and an 8 m by 5 m meeting room.

While many different experiments were performed in these environments, we will highlight two in particular. First of all, One of the earliest aspects which the authors investigated was the actual impact of system noise (i.e., deviation from the trained path) on the identification accuracy of a purely RF CSI-based approach. For a candidate group size of six humans, it was shown that the performance decreased from 79% when the subject walked alongside the predefined path to 45% when the subject was on average 0.8 m removed from this path. This clearly illustrated the magnitude of this issue.

Second, in order to evaluate if their multimodal system could alleviate the problem, they compared the performance of Rapid to a purely RF CSI-based approach and to a purely audio-based approach. The algorithms were applied to a noisy dataset collected in all three environments. System noise was introduced due to the fact that subjects were instructed to walk freely in a certain direction and not follow a specific predefined path. Additionally, environmental noise (audio noise) was generated as well in order to investigate its influence on the acoustic modality. For all environments and all possible candidate group sizes ranging from 2 to 8, the Rapid system outperformed the separate radio-based and audio-based systems. The differences in identification accuracy were much less pronounced, however, in the corridor environment. This was interpreted by the authors as a result of how narrow this specific environment was. Due to the fact that the corridor was only 2 m wide, there was less opportunity for a subject to deviate too strongly from the training path. Therefore, this observation seemed to strengthen the validity of the Rapid multimodal approach.

An overview of the most important RF-based identification techniques which we discussed in this section is provided in Table 7.

## 5. Future Research Directions

In this section, we will discuss several important future research directions we have identified after reviewing the available literature. This is by no means meant to be a comprehensive list of each and every potential technical innovation but is rather a collection of existing, high-level issues within the domain of DFL whose resolution could provide a major boon towards the further development of the field. Some of these issues have been touched upon in the previous sections and our viewpoint regarding these matters will be explained in more detail here.

### 5.1. Publicly Available Datasets

A major shortcoming within the current state of the art which we explicitly want to draw attention to is the lack of publicly available datasets. In the vast majority of the research papers, we discussed previously, the authors constructed their own RF network in one or more specific environments, defined and performed a set of experiments and applied their algorithms on the obtained measurement data. Situations in which data was reused from other papers were rare and raw measurements were often not made available to the wider research community.

A major exception to this can be found in the measurements that were obtained during DFL research performed by members of the SPAN (Sensing and Processing Across Networks) Lab at the University of Utah. Several of their datasets (and additional resources such as implementations of certain algorithms) can be found at [132]. The reuse of this data has already been remarked upon in previous sections. For example, the measurements performed by Wilson et al. for VRTI through-wall experiments in [57] were later reused by Zhao et al. in [60,67] for their SubVRT and KRTI research. In this case, all three publications were co-authored by professor Patwari from (at the time) the University of Utah. The outdoor dataset collected by Wilson and Patwari for shadowing-based RTI [18], however, was reused by an entirely different team of researchers from the Spanish Centre for Automation and Robotics CSIC-UPM in [64]. Similarly, Lei et al. [123] from Wuhan University reused the passive fingerprinting data Mager et al. [122] collected for their research regarding database longevity.

As stated before, these examples are clearly still the exception rather than the rule. We find this to be regrettable because we believe that the more common use of public datasets would provide numerous advantages to this field of research. First of all, widespread availability of DFL measurement data performed in a multitude of different environments would mean that the construction and installation of an RF network would no longer be necessary for researchers who wish to focus purely on algorithmic aspects. As an additional advantage, this could potentially lower the barrier of entry into the field and aid in popularizing DFL research in general. Second, realistic comparisons between different techniques in different types of environments would become much more feasible. Currently, direct comparisons between techniques only tend to occur if a researcher wishes to compare a newly proposed technique to one or more state-of-the-art approaches. These approaches are then applied to the RF measurements the researcher collected, after which the resulting target detection, tracking and/or identification accuracies often demonstrate the superior performance of the newly proposed technique. Even if precautions are taken in order to ensure a fair comparison, however, the techniques are still only applied to measurements performed by the inventors of the newly proposed technique in a limited number of environments. The current difficulty in comparing different techniques has been noted previously within the field, most notably in a recent call for participants for a device-free localization competition at CPS-IoT Week 2019 [133]. Third, the use of measurements from a vast array of different setups would make it much easier to identify potential weaknesses and strengths of a technique for different types of environments, hardware, frequency bands, RF sensing modalities and experimental scenarios in general. This allows for more objective comparisons of the suitability of different systems in different applications. Finally, we also believe that the common use of publicly available datasets will strongly encourage cooperation between DFL researchers worldwide. Data reusability is an increasingly important topic within scientific fields in general and the public sharing of datasets is encouraged by the existence of online repositories such as the UCI Machine Learning Repository [134]. Additionally, in the related field of tagged localization, many different resources have been made publicly available [135,136,137] as well. We believe DFL should follow this evolution.

### 5.2. Deployment in Complex Environments and Associated Constraints

Another important future research direction is the translation of experiments ‘in the lab’ to more realistic setups which need to take certain constraints into account. Within the context of tracking, a tactical RTI study performed by Maas et al. [138] is a good example of this concept. They investigated the use of a fade-level RTI-system based in the context of tactical operations performed by special operations forces (SOF). The feasibility of rapid system deployment, node self-localization, and online (re)calibration measurements were all practical aspects that the authors focused upon in this study. Furthermore, the potential advantages of using (circularly polarized) directional antennas instead of omnidirectional antennas in these types of scenarios were looked at as well. (This aspect was but a small sidetrack of this paper and the measured accuracy improvements were comparatively limited. However, it should be noted that later research papers do exist which focus entirely on this aspect and which strongly indicate the importance that antenna design could have on the performance of actual tactical systems [139,140].) Finally, the authors organized a live demonstration for members of a SWAT team and other law enforcement agencies and therefore had direct contact with potential end-users of these types of systems.

Similar constraints associated with long-term deployments primarily came to the fore during an actual experimental long-term deployment of an elderly monitoring system in [141]. The authors of this study focused on the use of a fade-level based multi-channel RTI system in the context of assisted living. The idea was to continuously track the location of an elderly individual and provide this information to caretakers. One interesting observation resulting from this experiment was the requirement for long-term deployments to make use of self-updating online calibration methods. This is due to the fact that RF propagation patterns can change significantly as a result of small changes in the environment over time. Another interesting study regarding the use of DFL in more realistic and cluttered environments was performed by Ruan et al. in [142]. They developed a Bayesian probabilistic framework that combined the information provided by an RSS-based DFL system and a set of sensors that measured human-object interactions (e.g., watching TV or opening a fridge door). In doing so, the authors focused on the advantages smart home environments could offer, rather than on the constraints.

Unfortunately, these types of publications are still in the minority. In regards to RF-based tagless detection systems—particularly those with a focus on crowd estimation—the general lack of realistic setups is arguably even more egregious. The potential use of these systems in crowd safety contexts has been discussed in multiple publications [46,49,50]. Nevertheless, to the best of our knowledge, no experiments have been performed in environments containing actual large crowds of hundreds or even thousands of individuals, with the sole exception of our own limited feasibility study in a single environment at a music festival [55].

Even when not considering any particular practical constraints, applying existing DFL techniques on measurements performed in more realistic environments might lead to surprisingly poor results. Some highly interesting research was performed in this context by Patra et al. in [143]. The authors of this paper investigated the performance of three existing RTI techniques (shadowing-based RTI, CDRTI and FLRTI) with 2.4 GHz Wi-Fi nodes in five different environments. The impact of many different environmental, setup and algorithm-related parameters on the accuracy was investigated and particular attention was paid to the influence of targets outside of the measurement area and of co-channel interference. Comparatively poor results led the authors to conclude that these techniques were not feasible for accurate and cost-effective localization of human targets in complex indoor environments. Similar research was performed by Konings et al. in [144]. They compared different types of device-free tracking techniques: imaging-based KRTI [66,67], passive radio mapping-based SCPL [117] and link-based Ichnaea [94]. Experiments were performed in (a section of) an auditorium environment of size 5 m by 5 m and a lab environment of size 4.8 m by 9.2 m. The accuracy of the aforementioned methods was investigated for both 6 and 20 2.4 GHz TI CC2530 Zigbee nodes being present, as well as for different movement patterns of a single target. Results indicated that this movement pattern had a major impact on the tracking accuracy for all three studied techniques. Furthermore, tracking performance was clearly not consistent across the entire environment. It was also shown that no singular approach was superior to all others in all performed tests. The authors repeatedly stressed that fair comparisons between different DFL approaches could only be made experimentally in the same environment with the same setup and target trajectories. We believe this will be greatly aided by an increased availability of public datasets, as we mentioned in the previous subsection.

We are of the opinion that a more pronounced focus on realistic setups is necessary for the maturation of this field of research and will eventually lead to a more widespread use of these types of technologies in actual commercial applications. Some steps towards the commercialization have already been taken; several patents have been filed based on RF DFL concepts, most notably by Wilson and Patwari [145]. Additionally, the company Xandem offers tagless localization kits based on radio tomographic imaging [146]. Nevertheless, commercialization is still rather limited and we hope that this will change over the course of the next decade.

### 5.3. Fingerprint Database Longevity

Compared to other DFL techniques such as RTI, passive fingerprinting approaches tend to require a surprisingly low amount of nodes to achieve accurate localization results. Unfortunately, the required regular manual recreation of the fingerprint database in order to adapt the system to environmental changes tends to make these approaches impractical for use in real-life applications. As mentioned before, this is an issue that has been discussed in existing literature, most notably by Xu et al. in [114], Mager et al. in [122] and Lei et al. in [123]. While several techniques have been proposed in these works to mitigate the problem somewhat and allow for longer-lived databases, this remains a fundamental problem which we believe acts as a roadblock in regards to further development of passive radio mapping-based techniques. Therefore, we are of the opinion that this issue should be one of the core focuses of future research performed within this field.

First of all, the full extent of the problem for different setups with different amounts and types of environmental changes needs to be investigated, which is once more an aspect that could greatly benefit from an increased amount of public datasets. Next, potentially interesting avenues to explore that might ameliorate this issue are the further development of fingerprint correction techniques as in [114] and a general increase of the fingerprinting accuracy through the use of more advanced fingerprint matching algorithms and multi-channel approaches as in [122]. The use of multiple frequency bands—a fairly young research topic in RF-based DFL in general—could be helpful here as well. Finally, another highly interesting approach would be to outright eliminate the manual construction of fingerprint databases and make use of automatically generated radio maps based on propagation models. A primitive version of this approach has been used in the context of identification, as we previously mentioned in our discussion of the WiDisc system [7].

### 5.4. Solving the Identification Problem

Determining certain target characteristics—especially actual identities in case of human targets—was already mentioned by Youssef et al. in their seminal work as a particularly difficult issue to resolve [6]. Indeed, only recently has progress been made towards this goal, with CSI-based gait analysis techniques receiving a significant amount of research attention [8,128,129,130]. While the steady progress with this technique is encouraging, further exploration of RSS-based systems like WiDisc [7] would be potentially interesting as well. While it is likely that one such system would never achieve the precision of a CSI-based approach and would only be able to differentiate between different body types, in and by itself this would provide interesting opportunities for enhancing existing RSS-based detection and/or tracking systems. Later in this section, we will discuss this in more detail.

### 5.5. DFL in next Generation Networks and IoT

The potential advantages of integrating device-free localization systems in an IoT context are regularly mentioned in literature [35,53,147]. We see the use of DFL techniques in an internet of things context as consisting of primarily two aspects: the practical implementation of a DFL system as part of a larger IoT ecosystem and the creation of a DFL system which makes use of ‘signals of opportunity’ from existing IoT communication infrastructure. The first aspect has previously been studied by Kianoush et al. in [148]. They designed an IoT-platform focused on passive radio sensing in general and experimentally evaluated its performance through two case studies, one focusing on assisted living applications and another on driver behavior recognition in a car. We encourage further research along this line, as integration within an IoT context is a highly practical topic that could increase future commercialization opportunities for DFL-based techniques.

The passive LTE-based crowd counting research performed by Di Domenico et al. [50] which we discussed previously is a good example of the second concept. Another interesting study in this context was performed by Savazzi et al. [149] and focused on the use of the built-in cellular radios in smartphones to track changes in the surrounding environment. The core sensing modality the authors used in this research was the cellular signal quality (CSQ). They developed a successful proof-of-concept in which body occupancy and movements of human entities could be detected and tracked in a confined space. Additionally, a case study in which the goal was to detect a human approaching a smartphone (in the experimental scenario seen as a potential tampering attempt) showed promising results as well. We believe that these types of studies are likely to become more numerous over time. An increasing amount of IoT networks are being installed in city environments worldwide and are already being utilized for active localization purposes [136]. These are all signals of opportunity which could be used for passive localization systems. The general concept of using existing network infrastructure to implement DFL solutions is not even particularly novel, given the fact that the use of existing Wi-Fi access points has been referenced before in literature [150].

One recent and highly interesting analysis which tackles both of the two aforementioned DFL-IoT aspects was performed by Savazzi et al. in [151]. In the context of (beyond) 5G technologies for next-generation industry, they identified two key enablers for future passive sensing technologies: the large-scale presence of smart antennas supporting software-defined beam steering and the consumer-level availability of extremely high frequency (EHF) RF-devices. In regards to beam steering, the authors illustrate its potential benefits with an example focused on body motion tracking. This example supposes a three-antenna Wi-Fi access point which can electronically steer the emitted 5 GHz RF-waves according to a set of beam steering profiles. By rapidly switching between two profiles which radiate in different directions while a human in motion is near the communication links between the AP and a set of receivers, body motion can more easily be isolated from environmental effects in the resulting CSI data. Furthermore, analyzing which specific beam profiles are more or less sensitive to certain motions could lead to much more precise localization and activity recognition approaches. The use of these beam steering profiles would, however, greatly increase the dimensionality of the CSI measurements. This will, therefore, require the development of suitable techniques which can successfully extract the desired information from these massive amounts of data, opening up an important new area of research within DFL. Furthermore, this data will likely be collected in large and complex IoT-networks consisting of many spatially diverse sensors. This means that the specific topology of these networks and the distribution of computing power (e.g., long-term analyses in the cloud, short-term analyses through edge computing) will be a highly important DFL-topic as well. Additionally, optimizing the deployment of all of these sensors would be a highly interesting subject of study as well. The channel charting approach proposed by Studer et al. [152] in which multi-antenna network elements can obtain a radio geometry chart of its surroundings, could be a potentially helpful tool in this context.

The potential value of ubiquitous EHF devices, the second key enabler identified by Savazzi et al., was illustrated with an industrial case study regarding very precise discrimination between different types of body motions. The scenario comprised the deployment of a 100 GHz radio source and a 32 × 32 detector array to implement a virtual fence. This fence was capable of differentiating between safe and unsafe human activities in close proximity to robotic manipulators.

Another potentially interesting IoT-related aspect which could also be a topic for future research is an in-depth comparison between the tracking accuracies of device-based and device-free localization approaches. One such comparison has already been studied by Zhao and Patwari in [153]. The authors implemented two tagged algorithms, one based on a standard maximum likelihood estimator (MLE) and one which incorporated the non-isotropic antenna gain pattern for the tag due to the influence of the human body. This second algorithm was called Alternating Gain and Position Estimation (AGAPE). Their tracking accuracies were experimentally compared to those of shadowing-based RTI, VRTI, and SubVRT for a single target in an outdoor, indoor and indoor through-wall environment. Surprisingly, the tagless techniques consistently outperformed the tagged techniques. The authors strongly suspected the high node densities of their experimental setups to be the cause of this. These results clearly indicate a need for further research. Do high-density setups consistently lead to tagless techniques outperforming tagged techniques in different types of environments? How can ‘high density’ be defined quantitatively? Are combinations between tagged and tagless systems feasible and/or useful? Are there current IoT contexts in which tagged solutions are used that could be replaced by tagless approaches? These are all interesting future research questions.

### 5.6. RSS to CSI

The vast majority of the techniques we have discussed over the course of this survey paper use either RSS or CSI as their RF-based sensing characteristic. Generally, CSI-based approaches have been developed more recently and tend to obtain a higher accuracy when compared to RSS-based methods. The vastly larger amount of information CSI provides regarding an RF link is the primary reason for these results. A highly interesting in-depth analysis regarding the differences between CSI and RSS(I) as it relates to indoor localization was made by Yang et al. [154] in 2013. While primarily focused on active localization, several RSS and (at the time very recent) CSI-based passive approaches were discussed as well.

Nevertheless, we are of the opinion that both methodologies still need to be developed concurrently, together with other potentially interesting sensing characteristics (e.g., raw waveforms analyzed by software-defined radios). The sensing characteristic that is most suitable can depend heavily on the specific application. For example, CSI-based approaches require the use of OFDM-based Wi-Fi and assume the availability of the necessary bandwidth, which might not always be the case.

### 5.7. Combining Detection, Tracking and Identification in a Single System

We have structured this survey paper according to the three main aspects of device-free localization: detection, tracking, and identification. In each section, we discussed several DFL techniques whose primary focus lay on the corresponding aspect. Nevertheless, many of the discussed techniques implicitly or explicitly combine multiple aspects within a single system. Detection and tracking are particularly convenient candidates for implementing together, as you first need to detect how many targets are present in an environment before you can attempt to estimate their positions. Some systems consider the number of targets to be known, but for other approaches, the name of the technique itself (e.g., sequential counting parallel localizing or SCPL [117]) already indicates that the combination of both aspects was present.

We would like to encourage research into the potential benefits of even more explicit combinations of existing techniques, however. Even in cases where a tracking technique implicitly detects the amount of targets present (e.g., a multi-tracking RTI system which counts the number of blobs in the attenuation image), the use of a separate crowd counting method could provide additional information which could lead to more accurate estimations. Additionally, such a system could potentially detect situations in which the maximum amount of targets that can be accurately tracked are currently present within the environment. This type of situation could then be handled in a desired manner by a high-level application on top of the system.

Furthermore, combinations between identification and full-fledged tracking systems are, to the best of our knowledge, currently non-existent. While we acknowledge that the comparatively early stage of development of the field of identification currently makes this difficult, we nevertheless believe that such a combination would provide interesting opportunities. For example, the passive RSS fingerprinting based WiDisc system [7] proposed by Scholz et al. was shown to be capable of differentiating between multiple (vastly different) body types. The body type estimated by one such system could potentially be used to adaptively update the parameters of a passive tracking model. Similarly, the characteristics of an identified target’s gait by a CSI-based approach could be used in this manner as well. Another potential advantage at an even higher level would be to use earlier observed movement patterns by a specific target to predict live movements by the same target. Many possibilities do exist and we strongly believe an increase of studies regarding explicit combinations between the three aspects of DFL would greatly enhance this field of research.

## 6. Conclusions

In this survey paper, we have discussed the evolution of RF-based device-free localization since the inception of the research field slightly over a decade ago. Impressive advancements have been made regarding the three core aspects of DFL: detection, tracking, and identification. Robust detection systems which make use of measurements from a very limited amount of communication links are able to accurately estimate the number of targets present in an environment. A wide variety of tracking solutions exist to determine the successive positions of targets in through-wall scenarios, potentially enabling the use of these technologies in emergency applications. Finally, several steps have been taken towards a solution for the difficult identification problem, with methods of differentiation based on human gait analysis being an active topic of research.

The development of CSI-based approaches has played an important role in realizing the aforementioned advances. While requiring the use of OFDM-based Wi-Fi and assuming the availability of the corresponding amount of bandwidth, the vastly larger amount of information this characteristic provides regarding an RF communication link has proven to be extremely beneficial for DFL. Furthermore, research efforts to tackle difficult roadblocks regarding issues such as the simultaneous tracking of multiple targets (particularly in the context of passive radio mapping), online model parameter estimation and rapid deployment of RF nodes in through-wall emergency scenarios have significantly enhanced the possibilities of DFL technologies. Nevertheless, there are still many more future improvements to be made in order for the field to fully mature. The identification problem is far from fully solved, research regarding crowd estimation in truly large-scale environments is extremely limited and the development of passive radio mapping solutions has hit a major roadblock in the form of limited fingerprint database longevity. Furthermore, the results from recent comparative studies seem to imply that the implementation of several existing DFL technologies in different, more realistic environments can lead to surprisingly poor accuracy results. Additionally, the commercialization of DFL, in general, has been limited thus far. In both a commercial as well as a research context, DFL is still relatively unknown within the wider community of RF-based localization.

We believe that there are several high-level research approaches that will aid the search towards solutions for the current main issues plaguing different domains within DFL. These approaches include a more pronounced focus on realistic test setups with their associated constraints and an increased amount of research regarding explicit combinations of multiple DFL aspects in a single system. The future research direction which we emphasize most of all, however, is the greatly increased availability of public datasets from a variety of different setups and measurement scenarios. Not only will this provide many more opportunities for researchers to experiment with and improve their techniques, it will also increase international cooperation between different research teams and cause the field of research, in general, to become much more known. It is our hope that this survey paper will contribute to the popularization of DFL as well.

## Figures and Tables

**Table 1:** Detection techniques.

Reference	Technique(s)	Standard(s)	# of Nodes	Detection/Counting/Classification	Environment	RSS/CSI	Reported Accuracy
Youssef et al. [6]	Moving Average-based Moving Variance-based Detection	IEEE 802.11b (2.4 GHz)	2 TX 2 RX	Detection only	Lab (idealized)	RSS	100% (0 false positives) 100% accuracy (0 false positives)
Moussa and Youssef [42]	Moving Average-based Moving Variance-based MLE	IEEE 802.11b (2.4 GHz)	2 TX 2 RX	Detection only	25 m^2^ lab	RSS	Recall: ≤0.8 and Precision: 0.2–0.3 Recall: ≤0.8 and Precision: 0.2–0.4 Recall: 0.9 and Precision: ≤0.2
Lee et al. [43]	Fluctuation Histograms	IEEE 802.15.4 (2.4 GHz)	1 TX 1 RX	Detection only	24 m^2^ meeting rooms	RSS	100% with min 2, 0 and 2 false positives in 3 rooms respectively, with 10 detectable human motion events
Kosba et al. [44]	RASID Moving Average-based Moving Variance-based MLE	IEEE 802.11b (2.4 GHz)	4 TX 3 RX	Primarily detection	186 m^2^ office floor two∼139 m^2^ home floors	RSS	F-measures Initial Experiment RASID: 0.96 (office) and 0.93 (home) M.A.: 0.86 (office) and 0.69 (home) M.A.: 0.86 (office) and 0.69 (home) MLE: 0.91 (office) and 0.94 (home) F-measures 2nd Experiment RASID: 0.94 (office) and 0.92 (home) M.A.: 0.84 (office) and 0.70 (home) M.V.: 0.74 (office) and 0.71 (home) MLE: 0.60 (office) and 0.65 (home)
Xiao et al. [45]	FIMD RASID-like	IEEE 802.11n (2.4 GHz)	1 TX 1 RX	Primarily detection	77 m^2^ lab narrow 48.75 m^2^ corridor	CSI RSS	FIMD ≥90% with 14% false positives (lab) ≥70% with ≤1% false positives (lab) ≥90% and 9% false positives (corridor) RASID detection rate very slightly below FIMD
Yuan et al. [46]	K-means clustering	IEEE 802.15.4	16 TRX	Classification 0-3,4-10,10+	324 m^2^ empty room	RSS	94% (static crowd) 86% (moving crowd)
Xi et al. [47]	FCC (Frog-Eye)	IEEE 802.11n (2.4 GHz)	1 TX 4 (max) RX	Counting up to 30 people	multiple indoor and outdoor environments	CSI	70% (error < 2, outdoor) 98% (error < 2, indoor)
Depatla et al. [48]	Minimizing Kullback–Leibler	IEEE 802.11g (2.4 GHz)	1 TX 1 RX	Counting up to 9 people	70 m^2^ outdoor 33 m^2^ indoor	RSS	96% (error < 2, omnidirectional, outdoor) 100% (error < 2, directional, outdoor) 63% (error < 2, omnidirectional, indoor) 100% (error < 2, directional, indoor)
Fadhlullah and Ismail [49]	Statistical Approach	Zigbee (2.4 GHz)	1 TX 3 RX	Classification low (5), medium (10 or 15)	100 m^2^ indoor	RSS	72.92%
Di Domenico et al. [50]	LTE signals of opportunity	LTE (796 MHz)	1 (LTE) TX 1 RX	Classification 0,1,2–3,4–5	45 m^2^ meeting room	RSRP	79%, 92% and 76% (depending on receiver location)
Cianca et al. [22]	Linear Discriminant Classifier	IEEE 802.11b (2.4 GHz)	1 TX 1 RX	Counting up to 5 people	30 m^2^ office 45 m^2^ meeting room	RRSS → CSI RSS	CSI: 72% (office) and 69% (meeting room) RSS: 70% (office) and 57% (meeting room)
Di Domenico et al. [51]	Trained-once Linear Discriminant Classifier	IEEE 802.11n/g (2.4 GHz)	1 TX 1 RX	Classification 0,1,2,3–4,5–7	Three meeting rooms: small (30 m^2^), medium (45 m^2^) and large (75 m^2^)	CSI	74% (small) 91% (error < 2, small) 52% (large) 81% (error < 2, large)
Di Domenico et al. [51]	Trained-once Bayesian Classifier on derived Doppler Spectrum	IEEE 802.11n/g (2.4 GHz)	1 TX 1 RX	Classification 0,1,2,3–4,5–7	Three meeting rooms: small (30 m^2^), medium (45 m^2^) and large (75 m^2^)	CSI	73% (small) 63% (large)
Zou et al. [53]	WiFree	IEEE 802.11n (5 GHz)	1 TX 1 RX	Counting up to 4, 7 and 11 people	14 m^2^ discussion room 35 m^2^ conference room 56 m^2^ seminar room	CSI	99.1% detection accuracy 92.8% counting accuracy
Denis et al. [55]	Probabilistic Neural Network	DASH7 (433 MHz and 868 MHz)	46 TRX	Classification Class 0–Class 6 Crowd sizes in 1000’s	1755 m^2^ indoor festival stage	RSS	>90% (error ≤ 1 category)

**Table 2:** Radio tomographic imaging techniques.

Reference	Technique(s)	Standard(s)	# of Nodes	Multitracking?	Environment(s)	Main Conclusions
Wilson and Patwari [40]	Shadowing-based RTI	IEEE 802.15.4 (2.4 GHz)	28 TRX	Limited	18.21 m^2^ open indoor	Shadowing-based RTI was shown to be a potential way of detecting human targets in an open test environment. No quantified accuracy was provided, however.
Wilson and Patwari [41]	Shadowing-based RTI	IEEE 802.15.4 (2.4 GHz)	28 TRX	Limited	Semi-open 40.97 m^2^ outdoor (containing trees)	The validity of the concept of Shadowing-based RTI was shown to hold true in a more complex outdoor environment containing trees. When comparing two idealized images based on a cylindrical human model with two attenuation images provided by the RTI algorithm, total squared errors of 0.021 (1 target) and 0.036 (two targets) were obtained.
Wilson et al. [57]	VRTI	IEEE 802.15.4 (2.4 GHz)	34 TRX	No	Indoor 72.46 m^2^ home (through-wall, most nodes outdoors)	0.63 m and 0.45 m average tracking errors for respectively a moving target and a semi-stationary target indicated the feasibility of VRTI. RTI-based approaches were therefore shown to be viable for through-wall scenarios which did not require empty environment calibration.
Zhao et al. [60]	SubVRT VRTI	IEEE 802.15.4 (2.4 GHz)	34 TRX	No	Indoor 72.46 m^2^ home (through-wall, most nodes outdoors)	SubVRT outperformed regular VRTI by 13.6% when using the same dataset as in [57]. When using a new dataset which contained large amounts of intrinsic motion caused by wind, subVRT outperformed VRTI by 40.5%, indicating the validity of the intrinsic—extrinsic motion concept.
Zhao et al. [67]	HD-RTI/KRTI VRTI SMC	IEEE 802.15.4 (2.4 GHz)	34 TRX	No	Indoor 72.46 m^2^ home (through-wall, most nodes outdoors) 60 m^2^ bookstore	The proposed KRTI technique was shown to be the—at the time—only real-time RTI technique capable of tracking both stationary and moving targets in both through-wall and non-through-wall scenarios without requiring any kind of training or empty room calibration.
Kaltiokallio et al. [68]	cdRTI PRR-multichannel RTI	IEEE 802.15.4 (2.4 GHz) (5 channels)	30 TRX	Very limited	Open 70 m^2^ indoor Complex lounge room (through-wall)	By utilizing multiple frequency channels, cdRTI became the first attenuation-based radio tomographic imaging technique capable of obtaining sub-meter accuracy in through-wall environments for stationary targets.

**Table 3:** Radio tomographic imaging techniques (2).

Reference	Technique(s)	Standard(s)	# of Nodes	Multitracking?	Environment(s)	Main Conclusions
Kaltiokallio et al. [62]	flRTI cdRTI	IEEE 802.15.4 (2.4 GHz) (4-5 channels)	30 TRX (open indoor) 33 TRX (apartment) 30 TRX (complex indoor)	No	Open 70 m^2^ indoor 58 m^2^ apartment Complex 70 m^2^ indoor (through-wall)	The experiments showed flRTI consistently outperforming cdRTI in regards to accuracy, even if the flRTI ellipse-width model was created based on measurements in a different environment. Additionally, the earlier observed feasibility of attenuation-based through-wall multi-frequency RTI systems was confirmed in this study as well.
Bocca et al. [63]	flRTI (servo-nodes) (4 channels)	IEEE 802.15.4 (2.4 GHz)	13 TRX (servo, apartment) 26 TRX (regular, apartment) 14 TRX (servo, lab) 28 TRX (regular, lab) 12 TRX (servo, office) 24 TRX (regular, office)	No	56 m^2^ apartment 54 m^2^ lab 100 m^2^ office space	The validity of the use of ’fade level’ as a concept within RTI was strenghtened by the results of this study, which showed reductions in localization errors between 30% and 37% when comparing servo-nodes to static nodes.
Bocca et al. [58]	Multi-tracking and multi-channel RTI	IEEE 802.15.4 (2.4 GHz) (4 channels in apartment,) 5 channels in open indoor and office)	30 TRX (open indoor) 33 TRX (apartment) 32 TRX (office)	Yes	Open 70 m^2^ indoor 58 m^2^ apartment Complex 67 m^2^ office	Multi-tracking RTI was demonstrated to be viable for up to four targets. The largest average tracking errors were observed in the complex office environment and were equal to 0.45 m, 0.46 m and 0.55 m for respectively 2, 3 and 4 targets being simultaneously present.
Adler et al. [70]	Shadowing-based RTI	Custom (cc1101) (868 MHz)	20 TRX	No	2 × 25 m^2^ open indoor	Results showed a maximum average localization error of 0.78 m for a single, stationary target (with Tikhonov regularisation), indicating the viability of sub-GHz frequencies for RTI.
Wagner et al. [75,76]	Passive RFID-based RTI	Bistatic UHF passive RFID (868 MHz)	36 transponders 4 reader antennas	No	Open 7.29 m^2^ indoor square	Within the inner environment surrounded by passive tags, a (real-time) mean localization error of 0.30 m was obtained. Localization could still occur outside this environment, with a mean localization error of 0.45 m.
Jimenez et al. [64]	Shadowing-based RTI (DFL-RTI) DFL-PF Tagged trilateration	Active RFID (433 MHz) IEEE 802.15.4 (SPAN-Lab) (2.4 GHz)	40 RFID tags 8 reader antennas 6 mobile tags/target 28 TRX (Span-Lab)	No	16 m^2^ indoor home 40.97 m^2^ semi-open outdoor (SPAN-Lab)	Results indicated both DFL approaches generally outperforming the tagged solution, with the RTI system being slightly more accurate than their own proposed solution. This demonstrated the viability of 433 MHz for RTI. The combination of DFL and the tagged system was only beneficial if the total amount of reader antennas was drastically reduced to 3.

**Table 4:** Radio tomographic imaging techniques (3).

Reference	Technique(s)	Standard(s)	# of Nodes	Multitracking?	Environment(s)	Main Conclusions
Fink et al. [73]	Shadowing-based multi-frequency RTI (redundant RTI)	IEEE 802.15.4 (2.4 GHz and 868 MHz)	14 ’nodes’: 28 TRX (2.4 GHz) 28 TRX (868 MHz)	Limited	Complex 52 m^2^ lab	The combination of both frequencies significantly outperformed the equivalent single-frequency system, showing the potential of using multiple frequency bands. Additionally, multi-tracking with the proposed system was shown to be somewhat feasible, but no quantifiable data was provided.
Denis et al. [71]	Shadowing-based single-frequency and multi-frequency RTI	DASH7 (433 MHz and 868 MHz)	20 TRX (868 MHz) 20 TRX (433 MHz)	No	Empty 60 m^2^ classroom	A multi-frequency RTI system was proposed which utilized only sub-GHz frequencies—433 MHz and 868 MHz. In 97% of cases, the presence of a single stationary target was correctly detected. The target could then be located with an RMSE of 0.54 m. This result strengthened the validity of the concept of multi-frequency RTI.
Denis et al. [74]	Shadowing-based multi-frequency RTI (variable ellipse λ)	DASH7 (433 MHz and 868 MHz)	37 TRX (868 MHz) (+ configurator and subcontroller) 37 TRX (433 MHz) (+ configurator and subcontroller)	Very limited	Complex 125 m^2^ 2-room office (connected by hallway)	Rather poor accuracy results (especially for multiple static targets) for both techniques suggested that the proposed systems were too naive for use in highly complex environments.
Kaltiokallio et al. [77]	ARTI flRTI	IEEE 802.15.4 (2.4 GHz) (4 channels in open indoor,) 4–16 channels in apartment, lounge)	30 TRX (open indoor) 33 TRX (apartment) 33 TRX (lounge)	No	70 m^2^ open indoor 58 m^2^ single bedroom apartment 86 m^2^ lounge room (through-wall)	The proposed adaptive RTI system could adaptively update its parameters while active. Regular ARTI doubled the median localization accuracy when compared to flRTI, while the addition of a smoothing method to train the model parameters led to a threefold increase.
Khaledi et al. [87]	Energy-efficient RTI (ellipse) Energy-efficient RTI (radius) Multi-channel shadowing-based RTI VRTI	IEEE 802.15.4 (2.4 GHz) (5 channels in open indoor, 4 channels in cluttered office, 1 channel in bookstore)	30 TRX (open indoor) 14 TRX (cluttered office) 34 TRX (bookstore)	No	70 m^2^ open indoor 52 m^2^ cluttered office 55 m^2^ bookstore	The newly proposed methodologies caused an energy usage decrease between 50% and 80% without having any negative impact on accuracy. The radius-based approach even caused a limited accuracy increase.

**Table 5:** Non-RTI model-based tracking techniques.

Reference	Technique(s)	Standard(s)	# of Nodes	Multitracking?	Environment(s)	Main Conclusions
Li et al. [89] (simulations) Chen et al. [90] (experiments)	SMC Shadowing-based RTI	IEEE 802.15.4 (2.4 GHz)	24 TRX	No	2 × Open 49 m^2^ outdoor (one containing tree)	The proposed DFL algorithm which made use of a particle filter (or sequential Monte Carlo method (SMC)) managed to obtain impressive accuracy-related results (0.49 m RMSE tree, 0.32 m RMSE treeless) when estimating the location of a single target. It consistently outperformed shadowing-based RTI in a direct comparison (0.86 m RMSE tree, 0.64 m RMSE treeless). Additionally, the second paper incorporated a methodology to successfully estimate unknown node locations.
Wilson et al. [91]	Fade-level Skew-Laplace Strength Model for DFL	IEEE 802.15.4 (2.4 GHz)	34 TRX	Yes	Bookstore Home (through-wall)	The proposed non-imaging DFL technique obtained impressive average localization accuracies for both single and double targets in both environments. Only when simultaneously tracking two moving targets in the through-wall home environment was the average localization error higher than 1 m.
Zheng and Men [92]	RSS model based on foreground detection	IEEE 802.15.4 (2.4 GHz)	24 TRX	No	Rectangular indoor room (through-wall)	An RMSE of 0.13 m was obtained when attempting to track a single moving target within the test environment. This clearly highlighted the potential of this technique which did not require the use of any type of offline training or calibration whatsoever.
Zhang et al. [93]	RASS	IEEE 802.15.4 (2.4 GHz) (multiple channels, 1 channel/hexagon) + Custom (Mica2 sensor board) (868 MHz)	3 TRX/triangle 10TRX (total)	Limited (different triangles)	Open 400 m^2^ indoor	Within a single triangular setup with the nodes being spaced 4 m part on the ceiling, an average localization error of 1.13 m was observed for a single moving target. Given the scalability of this technique, this was an impressive result which clearly demonstrated its potential.
Kaltiokallio et al. [101]	three-state RSS model SMC (EM) Exponential Rayleigh model	IEEE 802.15.4 (2.4 GHz)	1 TX 3 RX	No	3 corridors of widths 2.0 m, 3.0 m and 3.5 m	The proposed three-state model consistently outperformed the other techniques in the corridor environments, showing the validity of this approach.
Wang et al. [96]	Diffraction-based model Elliptical model Exponential Rayleigh model	IEEE 802.15.4 (2.4 GHz)	8 TRX	Yes	12.39 m^2^ indoor hallway	The proposed methodology narrowly outperformed both the elliptical-model based approach and the exponential rayleigh-based approach when attempting to track two moving targets within the environment. RMSEs of 0.11–0.12 m were obtained for the diffraction-based approach, 0.20–0.18 m for the elliptical model and 0.17–0.15 m for the exponential Rayleigh model.
Wang et al. [106]	BGA Fade-level Skew-Laplace CS-RTI	IEEE 802.15.4 (2.4 GHz)	16 TRX (+1 central node)	No	Open 64 m^2^ outdoor square	BGA significantly outperformed the other two algorithms both in regards to accuracy (0.155 m, 0.443 m and 0.322 m average localization error for BGA, fade level and CS-RTI respectively) as to the required running time when tracking a single moving target.
Hillyard and Patwari [103]	MLL HMML RTI KRTI LDA	IEEE 802.15.4 (2.4 GHz)	20 TRX (classroom) 32 TRX (home) 15 TRX (basement)	No	Open Classroom Furnished floor of home Furnished basement	Both the MLL and HMML approaches reduced the localization error between 11% and 51% when compared to the other methodologies. Furthermore, this performance did not degrade as a result of both deliberate and non-deliberate changes made to the environment.

**Table 6:** Passive fingerprinting techniques.

Reference	Technique(s)	Standard(s)	# of Nodes	Multitracking?	Environment(s)	RSS/CSI?	Reported Accuracy
Youssef et al. [6]	RSS histogram fingerprinting (Bayesian)	IEEE 802.11b (2.4 GHz)	2 TX 2 RX	No	Lab (idealized)	RSS	86.3%–89.7% fingerprint matching accuracy 0.21–0.16 m mean error (depending on training set)
Seifeldin and Youssef [113]	Nuzzer (Bayesian) Deterministic Random	IEEE 802.11b (2.4 GHz)	3 TX 2 RX (large office) 2 TX 3 RX (small office) 3 TX 4 RX (corridor)	Very Limited	1500 m^2^ office 130 m^2^ office 24 m^2^ curved corridor	RSS	2.9 m median error (Nuzzer, large office, discrete) 8.4 m median error (MDE, large office, discrete) 14 m (Random, large office, discrete) 1.82 m (Nuzzer, large office, continuous) 0.85 m (Nuzzer, small office, continuous) Multi-tracking shown to be potentially feasible
Xu et al. [114]	LDA MED QDA	Custom (250 kbps, MSK-modulated) (433.1 MHz and 909.1 MHz)	8 TX 8 RX 13 TX 9 RX	Limited (# of targets known)	40 m^2^ apartment 150 m^2^ office	RSS	90.1%—0.44 m median error (LDA, apartment) 81.7%—0.55 m median error (MED, apartment) 81.1%—0.53 m median error (QDA, apartment) 93.8%—1.4 m median error (LDA, office) 83.5%—0.89 m median error (LDA, apartment, 3 targets) 90% (LDA, apartment, 1-month old database, truncated correction)
Xu et al. [117]	SCPL	Custom (250 kbps, MSK-modulated) (909.1 MHz)	13 TX 9 RX 13 TX 9 RX	Yes	150 m^2^ office 400 m^2^ open indoor	RSS	84%—1.08 m average error (office) 86%—1.49 m average error (open indoor)
Sabek et al. [120]	ACE Nuzzer SCPL SPOT	IEEE 802.11(?) (exact 802.11 protocol not specified)	2 TX 3 RX	Yes	114 m^2^ apartment 130 m^2^ office	RSS	2.11 m median error (ACE, apartment, 1–3 targets) 1.44 m median error (ACE, office, 1–3 targets) 2.42 m median error (SCPL, apartment, 1–3 targets) 1.61 m median error (SCPL, office, 1–3 targets) 2.54 m median error (SPOT, apartment, 1–3 targets) 1.75 m median error (SPOT, office, 1–3 targets) 2.77 m median error (Nuzzer, apartment) 1.63 m median error (Nuzzer, office)
Mager et al. [122]	KNN LDA SVM Random Forests	IEEE 802.15.4 (2.4 GHz) (8 channels)	30 TRX	No	84 m^2^ home	RSS	47.9% error rate (KNN) 16.1% error rate (LDA) 8.3% error rate (SVM) 3.5% error rate (Random Forests) 0.185% error rate (random forests channel optimization)
Xiao et al. [109]	Pilot RASID Nuzzer	IEEE 802.11n	2 TX 2 RX	No	77 m^2^ lab L-shaped 776 m^2^ lobby	CSI	Accuracies up to 98% (Pilot, lab) 90% detection rate (Pilot, false positives ≥ 30%, lab) < 80% detection rate (RASID, false positives ≥ 30%, lab) Similar obeservations in lobby environment (slightly worse localization accuracy)

**Table 7:** Human identification techniques.

Reference	Technique(s)	Standard(s)	# of Nodes	Set Size	Strangers?	Static/Moving	Environment(s)	RSS/CSI	Reported Accuracy
Scholz et al. [7]	WiDisc	IEEE 802.15.4 (2.4 GHz)	4 TRX	3	No	Static	21.53 m^2^ lab	RSS	67% (Simulated fingerprints) 76% (Measured fingerprints)
Zhang et al.[128]	WiFi-ID	IEEE 802.11n (5 GHz)	1 TX 1 RX	2–6	No	Moving	indoor corridor	CSI	93% (set size 2) — 77% (set size 6)
Zeng et al.[129]	WiWho	IEEE 802.11n	1 TX 1 RX	2–6	Yes	Moving	3 indoor environments	CSI	92% (set size of 2) — 80% (set size 6)
Xin et al.[130]	FreeSense	IEEE 802.11n (2.4 GHz)	1 TX 1 RX	2–6	No	Moving	30 m^2^ smart home	CSI	94.5% (set size 2) — 88.9% (set size 6)
Lv et al.[131]	Wii	IEEE 802.11n	1 TX 1 RX	2–8	Yes	MovingMoving	20 m^2^ meeting room	CSI	98.7% (set size 2) — 90.9% (set size 8)
	WiWho	IEEE 802.11n	1 TX 1 RX	2–8	Not used	Moving		CSI	90%–95% (set size 2) — 65%–70% (set size of 8)
	Freesense	IEEE 802.11n	1 TX 1 RX	2–8	No	Moving		CSI	90%–95% (set size 2) — 75%–80% (set size 8)
Chen et al. [9]	Rapid	IEEE 802.11(?) (not specified)	1 TX 1 RX	2–6	Yes	Moving	2 m-wide corridor 54 m^2^ lab 40 m^2^ meeting room	CSI + Acoustic	92% (set size 2) - 82% (set size 6)
